# Solar-driven upcycling of plastic waste using plasmonic black gold

**DOI:** 10.1039/d5sc08424e

**Published:** 2025-12-23

**Authors:** Saideep Singh, Gunjan Sharma, Mamata Joshi, Vivek Polshettiwar

**Affiliations:** a Department of Chemical Sciences, Tata Institute of Fundamental Research Mumbai 400005 India vivekpol@tifr.res.in

## Abstract

Plastic waste accumulation poses a pressing environmental challenge, calling for sustainable routes to convert it into value-added products under mild conditions. Conventional Lewis acid-mediated upcycling relies on hydride transfer and carbocation formation but requires ionic liquids to stabilize intermediates and sacrificial alkylating agents like isopentane to overcome thermodynamic barriers. Here, we present a solar-driven, sacrificial-agent-free approach for catalytic plastic upcycling using plasmonic black gold nanostructures. Under visible-to-NIR irradiation, black gold activates *tert*-butyl chloride (TBC) through combined photothermal and hot-electron driven activation, generating reactive carbocations in polymer chains while converting Al_2_Cl_6_ into catalytically active AlCl_3_*in situ*. This dual activation eliminates the need for ionic liquids and isopentane, enhancing both efficiency and sustainability. The system achieves >80% plastic conversion within one hour solely by light illumination with >75% selectivity toward C_6_–C_10_ alkanes. Mechanistic studies confirm that plasmonic excitation promotes TBC dissociation and sustains AlCl_3_ generation throughout the catalytic cycle. The catalyst exhibits excellent recyclability over multiple cycles without loss of activity. A proof-of-concept outdoor experiment under natural sunlight further validates its real-world applicability. This work represents a unique demonstration of plastic upcycling powered solely by sunlight using a plasmonic catalyst, merging broadband light harvesting, hot-carrier chemistry, and Lewis acid catalysis into a unified, sustainable platform for decentralised upcycling of plastic waste.

## Abstract 2

Sustainable plastic waste upcycling using plasmonic black gold achieves >80% conversion and >75% alkane selectivity in 1 hour. Driven by sunlight, this sacrificial-agent-free method uses photothermal heating and hot electrons to create fuels.

## Graphical abstract

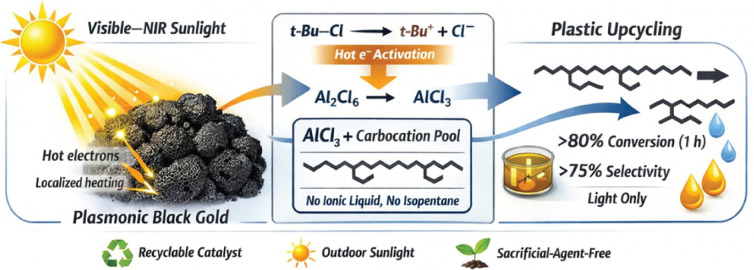

## Introduction

The global accumulation of plastic waste poses a growing environmental and resource challenge. These polymers are chemically less reactive, thermally stable, and widely used, making their recycling particularly difficult.^1–4^ Traditional recycling methods (mechanical reprocessing, pyrolysis, *etc.*) often suffer from high energy use, limited product value, and downcycling of material properties. On the other hand, chemical upcycling offers a pathway to transform waste plastics into valuable fuels and fine chemicals. However, current catalytic upcycling strategies often require harsh conditions, including high temperatures, pressurised hydrogen, reactive solvents, or complex co-reactants, limiting their environmental and economic viability.^1–7^

A notable breakthrough was recently reported,^8,9^ where the authors introduced a tandem catalytic system using a Lewis acidic ionic liquid in iso-pentane (iC_5_) to convert polyolefins into liquid alkanes at ∼70 °C. Their system cleverly couples C–C bond cleavage with *in situ* alkylation, enabling efficient low-temperature upcycling. However, despite its ingenuity, the method still depends on (i) external thermal input, (ii) specific hydrocarbon co-reactants (iC_5_), and (iii) ionic liquids, which are expensive, corrosive, and difficult to scale.

Photocatalytic upcycling of plastic waste has emerged as a promising strategy to upcycle polymers under mild conditions using solar energy.^10–14^ A variety of innovative photocatalytic systems have been reported for the upcycling of plastic waste, reflecting remarkable progress in this emerging field. Semiconductors such as MoS_2_/g-C_3_N_4_ heterojunctions,^15^ CN_*x*_|Ni_2_P,^16^ ultrathin Nb_2_O_5_ layers,^17^ and vanadium-based oxides^18^ have demonstrated efficient charge separation and light harvesting. Organic photocatalysts, including BrCH_2_CN-thioxanthone,^19^ anthraquinone,^20^ and phenothiazine derivatives,^21^ have offered elegant metal-free strategies, while hybrid and composite systems, such as CdS/CdO_*x*_ quantum dots,^22^ sulfur-vacancy-rich CdS,^23^ Co-Ga_2_O_3_,^24^ Zr-doped CoFe_2_O_4_ quantum dots^25^ and iridium complexes,^26^ have shown excellent activity and selectivity. Furthermore, enzyme-assisted platforms like TiO_2_|CotpyP^27^ and single-atom catalysts like M_1_-TiO_2_  ^28^ highlight the diversity of mechanistic approaches being pursued. Acid-mediated photocatalysis using AlCl_3_^29^ or pTsOH^30^ and FeCl_3_ ^31^ has provided compelling routes to oxygenates and monomers under mild conditions. Photothermal catalysis employing systems like Ni-TiO_2_-Al_2_O_3_,^32^ Ru-TiO_2_,^33^ TiO_2_-DEG,^34^ carbon quantum dots,^35^ and Cu/2D silicon in ionic liquid^36^ provides a complementary route for plastic upcycling, where light energy is harnessed to generate localized heating and promote bond cleavage.

Despite these significant achievements, many of these systems rely on additives such as sacrificial electron donors, alkylating agents, expensive solvents, ionic liquids or hydrogen gas. Others require elevated temperatures or UV light, which can limit sustainability and scalability. Thus, while the current body of work has laid a strong foundation, there remains a need for photocatalytic strategies that enable selective alkane generation from plastics using only solar light, without external heating, sacrificial reagents, or expensive solvents such as ionic liquids.

In this context, we pursued a distinct strategy, in favour of a purely sunlight-driven approach enabled by localised surface plasmon resonance (LSPR)-mediated photocatalysis.^37–48^ Building on our prior work in designing plasmonic nanocatalysts with strong light-harvesting and catalytic capabilities,^49–57^ we here report the upcycling of polyolefin plastic waste without external heating using plasmonic black gold^51–54^ and AlCl_3_ Lewis acid sites. “Black gold” consists of uniformly distributed gold (Au) nanoparticles on dendritic fibrous nanosilica (DFNS) and exhibits broadband localised surface plasmon resonance (LSPR), enabling efficient harvesting of solar energy from the visible to near-infrared (NIR) region.^51–54^ It generates energetic hot electrons that aid the formation of AlCl_3_ from Al_2_Cl_6_, facilitate carbenium ion formation and sequential C–C bond scission in polyolefins. The system operates without ionic liquids, without any sacrificial hydrocarbons like iC_5_, or any external thermal energy input. It facilitates the selective production of hydrocarbons and fine chemicals from waste plastics. Mechanistic studies support significant contribution of non-thermal activation pathways along with photothermal effects, wherein plasmon-induced hot electrons promote Lewis acid site-mediated cracking of polyolefins with high efficiency.

## Results and discussion

### Synthesis and characterization of broadband black gold nanostructures

**Fig. 1:**
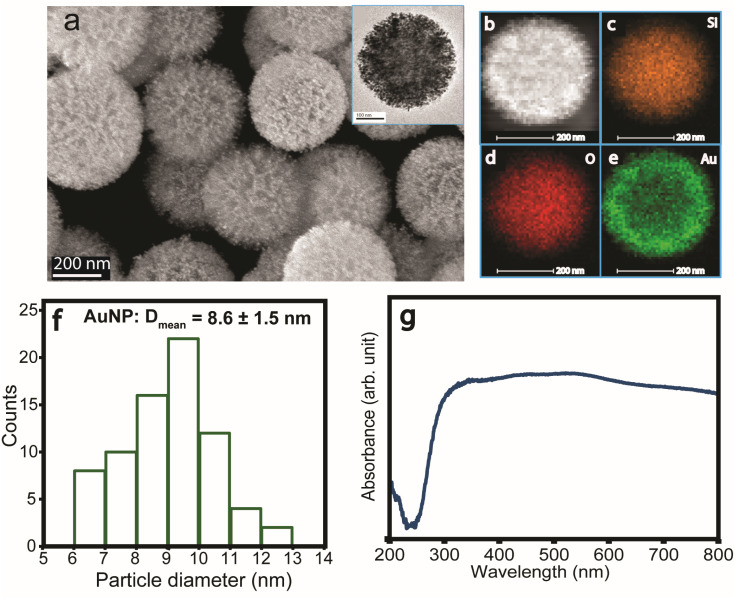
Characterization of dendritic plasmonic colloidosomes (black gold). (a) SEM images of DPCs; inset: TEM image of DPC; (b–e) HAADF-STEM image of DPCs and the corresponding elemental mapping; (f) particle size distribution of Au nanoparticles in DPCs and (g) UV-Vis diffuse reflectance spectra of DPCs showing broadband absorption.

Dendritic plasmonic colloidosomes (DPCs), also referred to as black gold, were synthesized following our previously reported one-pot protocol.^52^ Comprehensive structural and compositional analyses were conducted to evaluate their morphology and plasmonic architecture (Fig. 1). Scanning electron microscopy (SEM) and transmission electron microscopy (TEM) (Fig. 1a) show fibrous nanosilica spheres (∼400 nm) of DFNS uniformly loaded with gold nanoparticles (Au NPs), with an average particle size of ∼8.6 nm (Fig. 1f). Energy-dispersive X-ray spectroscopy (EDS) mapping confirmed a uniform gold loading (48 wt%), homogeneously distributed across the dendritic silica support (Fig. 1b–e and Table S1).

X-ray diffraction (XRD) verified the formation of metallic Au phases (Fig. S1). Nitrogen sorption analysis indicated a high surface area (192 m^2^ g^−1^) and a pore volume of 0.23 cm^3^ g^−1^ (Fig. S2). The synthesis process involved controlled nucleation and growth of Au NPs on high surface area dendritic fibrous nanosilica, yielding a heterogeneous distribution of interparticle distances and particle sizes. This variability enables black gold to achieve broad-spectrum light absorption, spanning the visible to near-infrared (NIR) range (Fig. 1g) by virtue of the plasmonic coupling. The coexistence of diverse particle sizes and plasmonic coupling among Au NPs induces localized electric field hotspots, which can potentially enhance their catalytic efficiency during waste plastic upcycling.

### Plasmonic upcycling of polyolefins

To study the solar-driven plasmonic upcycling of waste plastic, the photocatalytic reactions were conducted in a glass reactor under simulated solar illumination using a xenon lamp (400–1600 nm, 1.6 W cm^−2^, Fig. S3). The catalytic process consisted of black gold as a plasmonic light harvester, *tert*-butyl chloride (TBC) as a source of tertiary carbenium ions to initiate the chain reaction, and Al_2_Cl_6_ as a precursor for generating AlCl_3_ Lewis acid sites that facilitate hydride transfer. Due to photothermal and non-thermal effects (electric field enhancement and hot electron injection), plasmonic black gold was hypothesised to activate the C–Cl bond in TBC,^55^ to aid the generation of [Al_2_Cl_7_]^−^, which subsequently forms AlCl_3_, and also to activate C–H and C–C bonds for efficient hydride transfer and β-scission, respectively, during the cracking cycle.^58^

**Fig. 2:**
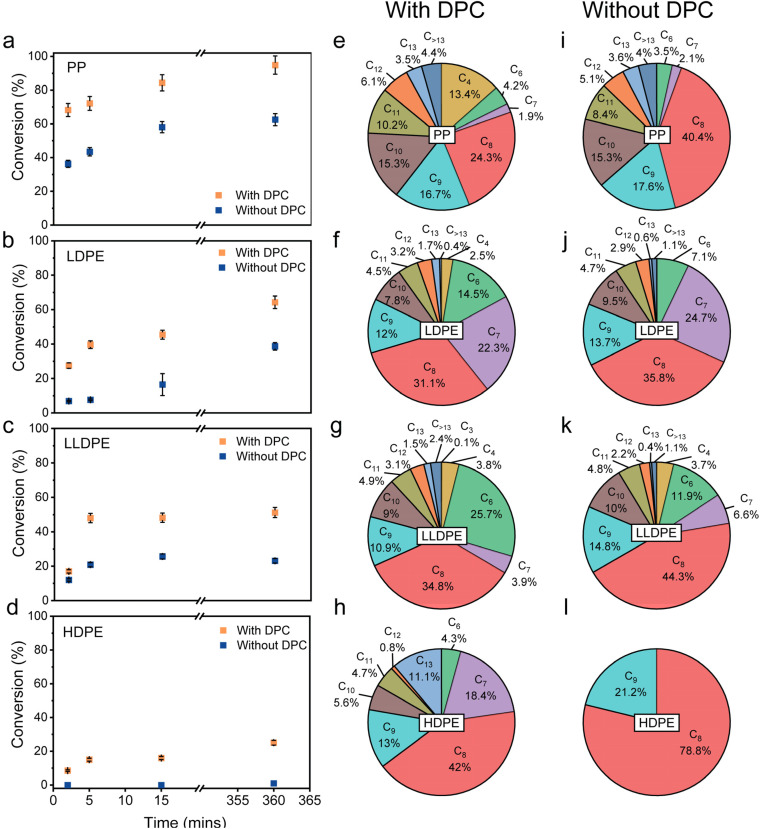
One-pot catalytic upcycling of different types of plastics into liquid alkanes. Time-dependent conversion profile of (a) PP, (b) LDPE, (c) LLDPE and (d) HDPE in the presence of an alkylating reactant (iC_5_) and the corresponding product distribution (excluding C_5_) of tandem cracking-alkylation of different plastic waste with (e–h) and without DPCs (i–l). Reaction conditions: 0.2 g PE/PP, 3 mL DCM, 1.29 mL iC_5_, 0.3 mmol Al_2_Cl_6_, 1.0 equivalent TBC, 30 mg DPC, under light of ∼1.6 W cm^−2^ (400–1600 nm).

Various polyolefin plastics were tested, including polypropylene (PP) recovered from discarded surgical masks and commercial grades of low-density polyethylene (LDPE), linear low-density polyethylene (LLDPE), and high-density polyethylene (HDPE). Initial tests employed isopentane (iC_5_) as an alkylating agent for olefinic scission products to thermodynamically favour the reaction by coupling the endothermic C–C bond cleavage with exothermic alkylation of olefins.^8,9^ Using iC_5_ as a sacrificial reactant poses challenges in terms of the origin and selectivity of the products. We later show that using DPC can help us avoid using iC_5_ altogether and still maintain high activity. In the initial tests, the standard reaction mixture contained one of the above-mentioned polyolefins (0.2 g), dichloromethane (DCM, 3 mL) as a solvent (recoverable), iC_5_ (1.29 mL), TBC as an initiator (32.4 µL), DPC (30 mg) and Al_2_Cl_6_. After degassing under vacuum, the mixture was irradiated under visible light (400–1600 nm, 1.6 W cm^−2^). We first optimised the amount of Al_2_Cl_6_ for PP conversion and found that 0.3 mmol (79.4 mg) was optimal, achieving quantitative conversion of 0.2 g of PP within 6 hours (Fig. S4). Using these optimized conditions, the upcycling of different polyolefins was evaluated with and without DPCs under light irradiation (Fig. 2).

The presence of DPCs significantly enhanced the conversion rate, nearly doubling the yield after 6 h compared to reactions without DPCs (Fig. 2a–d). The kinetics was also found to be faster in the case when DPCs were present, where most of the conversion occurred in the first 5 min of light irradiation. Furthermore, selectivity trends analysed after 6 h of irradiation showed that DPC-driven reactions favored the formation of lower alkanes (C_*n*_, *n* < 8, Fig. 2), attributed to more efficient carbenium ion generation and sequential C–C bond cleavage of higher chain hydrocarbons. As isopentane was used as the alkylating agent, the cumulative mass of final products was approximately double the initial polymer mass due to iC_5_ incorporation (Fig. S5), posing challenges in selectivity and yield calculations. The liquid products were extracted and analysed using gas chromatography-mass spectrometry (GC-MS). The product distribution (after subtracting C_5_ incorporation) was found to be mostly consisting of branched C_6_–C_10_ alkanes (∼85% for LDPE) (Fig. 2e–h, S6 and S7). Gas-phase analysis detected only trace propane (<0.1 wt%) and no methane or ethane (Fig. S8 and S9), indicating that the C–C bond cleavage predominantly proceeds through β-scission of carbenium ions rather than direct cracking of terminal carbon.

**Fig. 3:**
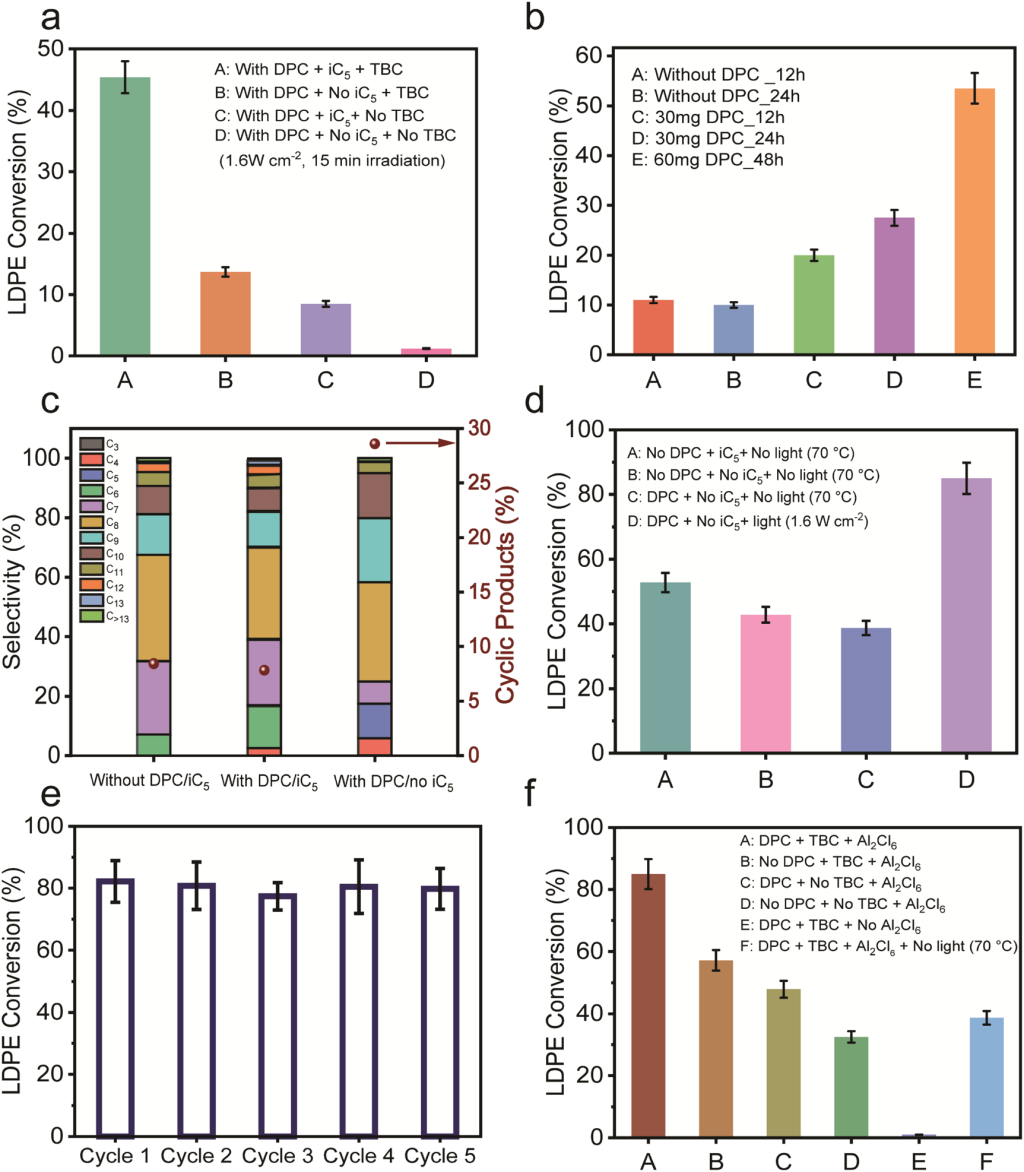
Plasmonic conversion of LDPE without the alkylating reactant (iC_5_). (a) Various control experiments showing the role of iC_5_ and TBC; (b) LDPE conversion in the absence and presence of DPCs and without iC_5_ under prolonged light irradiation; (c) comparison of product distribution of alkanes in the presence and absence of iC_5_ after 6 h; (d) comparison of LDPE conversion under thermal conditions (70 °C) and under photocatalytic conditions; (e) recycling test of DPCs in LDPE upcycling using the DPC/Al_2_Cl_6_/TBC system; (f) control experiments showing the role of different components, DPCs, Al_2_Cl_6_, TBC and light, after 60 min of illumination. Reaction conditions: LDPE (0.2 g for a–c and 0.1 g for d–f), 3 mL DCM, 0.3 mmol Al_2_Cl_6_, 1.0 equivalent TBC, 30 mg DPC, under light, ∼1.6 W cm^−2^ (400–1600 nm).

To probe the roles of iC_5_ and TBC, control reactions were conducted for LDPE conversion by sequentially eliminating each component (Fig. 3a). After 15 min of irradiation, significant decreases in conversion were observed when either iC_5_ or TBC was excluded, with near-negligible conversion in the absence of both. This demonstrated that while the DPC facilitated carbenium ion formation in the presence of TBC and AlCl_3_, it does not serve as an independent active site.

Further experiments were conducted, prolonging the light irradiation time to examine if isopentane could be eliminated altogether. In the absence of iC_5_ and DPCs, conversions remained low even after 24 h (Fig. 3b). However, in the presence of DPCs, conversion significantly increased with time, confirming DPC's role in sustaining the reaction even without the iC_5_ alkylating agent (Fig. 3b). Extending the reaction to 48 h with a higher amount of DPCs helped in achieving similar conversion levels as achieved with iC_5_. This highlights the intrinsic promotional role of DPCs in facilitating plastic upcycling, even in the absence of the exothermic alkylation pathway. Notably, in the absence of iC_5_, a substantial formation of lower alkanes (C_4_–C_5_) was observed, along with a notable increase in the production of C_8_–C_9_ cyclic hydrocarbons (∼28.6%) (Fig. 3c). In contrast, the presence of iC_5_ significantly suppressed cyclization, maintaining cyclic product selectivity below 5% due to the preferential alkylation pathway (Fig. S10).

To enhance the conversion even further without using iC_5_, the initial amount of LDPE was reduced to minimise competing reactive sites, leading to a substantial increase in conversion from approximately 25% to 85% (Fig. 3d and S11). Additionally, thermocatalytic reactions conducted at 70 °C (the typical post-illumination temperature measured *in situ*, Fig. S12 and S13) in the dark showed that light irradiation remains crucial as the conversion rates with DPCs in the dark were nearly half of those under illumination (Fig. 3d). This highlights a significant contribution of plasmonic non-thermal effects (electric field enhancement and hot electron injection) in DPCs along with photothermal enhancement in facilitating TBC activation to generate carbenium ions and successive chain scission as discussed in later sections.

DPCs demonstrated excellent reusability over five cycles with minimal loss of activity, retained morphology and minimal sintering (Fig. 3e, S14, S15 and Table S1), emphasizing their potential in sustainable plastic upcycling. Moreover, component-specific control experiments highlighted the necessity of each system component, *i.e.* DPCs, TBC, and Al_2_Cl_6,_ for optimal conversion efficiency (Fig. 3f). These results confirm the synergistic roles of plasmonic activation mechanisms and Lewis acid catalysis in orchestrating effective solar-driven upcycling of plastic waste.

### Uncovering the mechanism behind plasmon-driven LDPE conversion

**Fig. 4:**
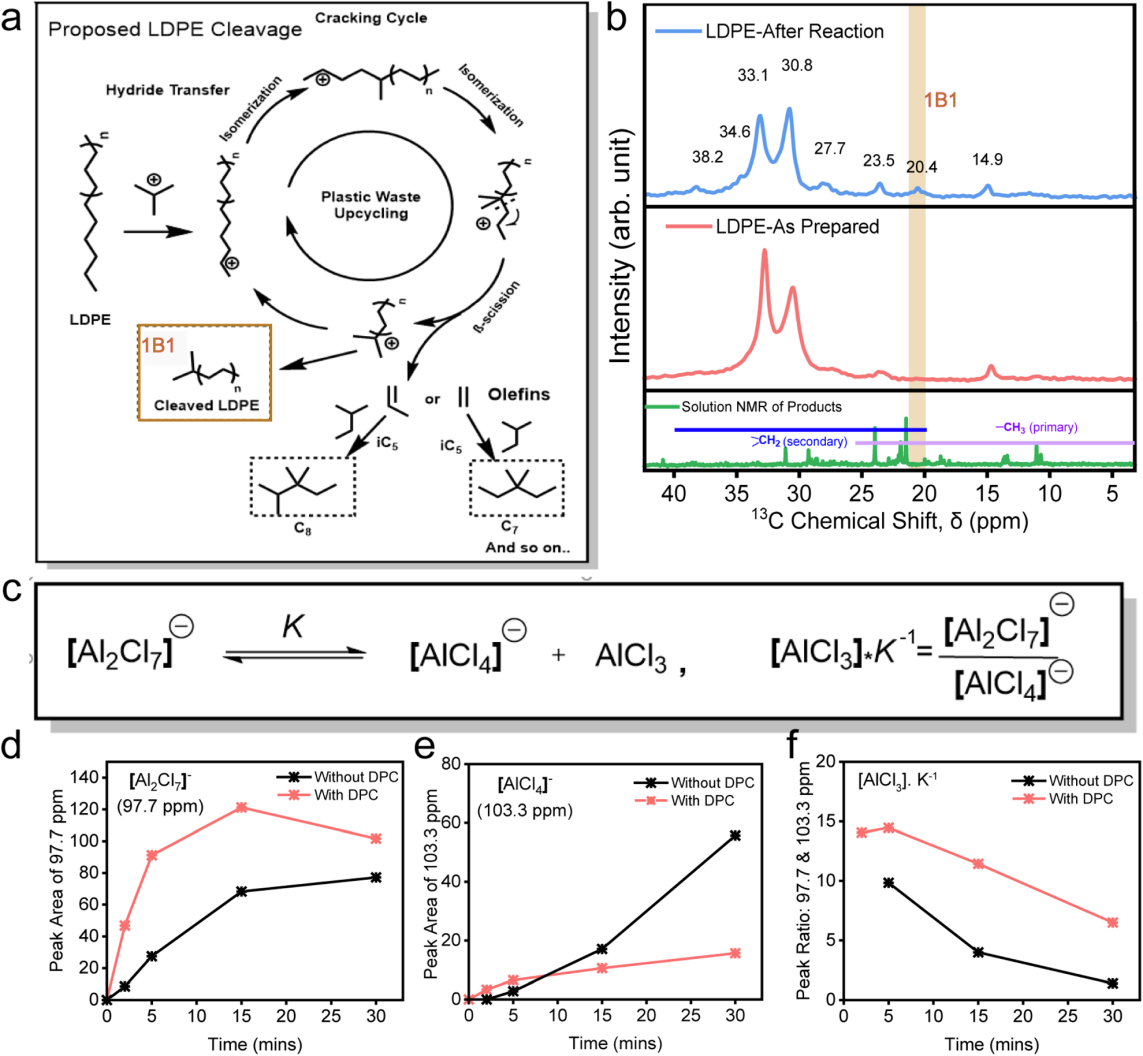
Elucidating the reaction mechanism. (a) Proposed LDPE cleavage in the plastic upcycling process; (b) ^13^C solid-state NMR spectra of solid LDPE particles before and after the reaction for the identification of cleaved LDPE, along with solution NMR spectra of products; (c) schematic of the formation of active AlCl_3_ species *via* dissociation of the dimeric [Al_2_Cl_7_]^−^ (K – equilibrium constant). Monitoring Al species at different time intervals with/without DPCs using solution state ^27^Al NMR spectroscopy, peak area of (d) [Al_2_Cl_7_]^−^ species, (e) [AlCl_4_]^−^ species and (f) their ratios at different light irradiation times.

Following the systematic evaluation of the individual roles of DPCs, TBC, and Al_2_Cl_6_ in facilitating the efficient conversion of LDPE, we propose a mechanistic pathway to elucidate their contributions to the upcycling process (Fig. 4). The AlCl_3_ Lewis acid is generated *in situ via* the interaction of Al_2_Cl_6_ with TBC, producing [Al_2_Cl_7_]^−^ and a *tert*-butyl carbenium ion, which initiates polymer cracking through hydride abstraction from the LDPE backbone (Fig. 4a). Lewis acidic AlCl_3_ promotes heterolytic C–Cl cleavage by polarizing the C–Cl bond, stabilizing the released Cl^−^ as AlCl_4_^−^/Al_2_Cl_7_^−^, as confirmed by ^27^Al NMR (Fig. 4d, e and S16–S19). This interpretation is supported by *in situ* DRIFTS, where the C–Cl stretching band (∼800 cm^−1^) exhibits a blue shift and reduced intensity, indicating progressive bond activation (Fig. S20). The resultant carbenium ion generated from TBC subsequently abstracts a hydride from the polyolefin backbone, thereby initiating chain cracking. Consistent with this pathway, the ^1^H NMR spectra exhibit a signal at ∼2.27 ppm, which can be assigned to the tertiary C–H proton of isobutane,^59^ a key intermediate formed after hydride abstraction. Notably, this signal is most intense for the DPC system (Fig. S21), reflecting more favorable reaction kinetics.

The plasmonic black gold catalyst enhances these steps through local field effects, hot-electron transfer, and photothermal heating,^53,55,60,61^ facilitating carbenium ion formation. Although plasmonic excitation may transiently generate radical species due to hot electron transfer, rapid charge relaxation to maintain Au neutrality renders a sustained radical pathway unlikely.

Upon initiation, the polyolefin chains form primary carbenium ions *via* hydride transfer, catalyzed by AlCl_3_,^8,9,58^ which are energetically unfavorable. These unstable intermediates undergo successive rearrangements, specifically hydride and methyl shifts, to yield more stable tertiary carbenium ions. These then undergo type-A β-scission, resulting in the formation of an olefin and a shorter carbenium ion.^62^ The resulting olefins can undergo either alkylation, in the presence of isopentane, to yield branched alkanes, or cyclization in its absence, thus driving the reaction forward (Fig. 4a and S10).

This mechanistic framework rationalizes the observed reactivity trend among various polyolefins. Polypropylene, characterized by methyl branches on alternating carbon atoms, facilitates the formation of tertiary carbenium ions and consequently exhibits the highest conversion. As the rate of β-scission increases with the branching degree, the trends observed for LDPE, LLDPE and HDPE could also be explained. Additionally, the more compact crystalline morphology of HDPE limits polymer–catalyst interactions, further reducing reactivity (Fig. 2).

To validate these mechanistic insights for polyolefin cleavage, we performed solid-state ^13^C NMR analysis on post-reaction LDPE solids (Fig. 4b). A new peak at 20.4 ppm was observed, which was absent in the spectra of liquid-phase products. This peak was previously assigned to β-methylene carbons adjacent to chain ends,^63,64^ indicating chain cleavage. ^13^C NMR peaks at 38.2, 34.6, 23.5, 14.9, and 11.2 ppm corresponded to tertiary and secondary carbons in the branched side chains and terminal methyl groups, while peaks at 33.1, 27.7 ppm corresponded to methylene groups of the main backbone.^65,66^ Interestingly, after the catalysis, the peak at 33.1 ppm, representing crystalline methylene (–CH_2_– backbone in LDPE), showed reduced relative intensity, suggesting depolymerization. Furthermore, the absence of methane and ethane in the gas phase indicates that cleavage proceeds predominantly *via* internal β-scission rather than terminal bond breaking. Importantly, the presence of DPCs plays a central role in this process by generating localized plasmonic ‘hot spots’ under visible light, which polarize and activate C–Cl, C–C and C–H bonds, facilitating the formation and propagation of carbenium ions. These reactive intermediates drive a sequence of hydride shifts, isomerization, and β-scission steps, efficiently breaking down polyolefin chains.

Having established a mechanistic framework involving carbenium ion formation, hydride transfer, and subsequent β-scission, we next sought to elucidate the role of *in situ* generated AlCl_3_ as a Lewis acid in promoting the reaction. To investigate the nature of the catalytically active aluminium species, solution-state ^27^Al NMR spectroscopy was conducted during the plasmonic reaction. This analysis enabled real-time monitoring of [Al_2_Cl_7_]^−^ and [AlCl_4_]^−^ species under light irradiation, both in the presence and absence of DPCs (Fig. 4d–f and S16–S19). AlCl_3_, the active Lewis acid species, is formed through chloride abstraction by Al_2_Cl_6_. When Al_2_Cl_6_ abstracts Cl^−^ from TBC, it produces [Al_2_Cl_7_]^−^ which subsequently dissociates and yields AlCl_3_ and [AlCl_4_]^−^ (Fig. 4c). While [Al_2_Cl_7_]^−^ has long been regarded as catalytically active,^67^ recent kinetic analyses by Zhang *et al.*^8^ showed that it is the transient AlCl_3_ formed from its dissociation that directly mediates catalysis. The relative concentrations of AlCl_3_ can be approximated from the ratio of [Al_2_Cl_7_]^−^ to [AlCl_4_]^−^ peak areas, scaled by the equilibrium constant (K^−1^) (Fig. 4c).

Under light irradiation, the presence of DPCs led to a consistently higher abundance of [Al_2_Cl_7_]^−^ and a corresponding decrease in [AlCl_4_]^−^ compared to reactions conducted without DPCs. This indicates that plasmonic effects in DPCs (thermal and non-thermal) accelerate the generation of Cl^−^ ions by efficient cleavage of the C–Cl bond in TBC, thereby promoting rapid conversion of Al_2_Cl_6_ to [Al_2_Cl_7_]^−^. In contrast, the reaction without DPCs exhibited a slower accumulation of [Al_2_Cl_7_]^−^ (Fig. 4d) and a gradual increase in [AlCl_4_]^−^ over time (Fig. 4e), suggesting a competitive conversion pathway in which the delayed availability of Cl^−^ facilitates the formation of catalytically inactive AlCl_4_^−^. The net effect is a significantly higher concentration of AlCl_3_ in the presence of DPCs throughout the reaction, aligning closely with the enhanced LDPE conversion observed in the presence of DPCs (Fig. 2 and 3). It should also be noted that the consequent decrease in the AlCl_3_ concentration could be due to the formation of an adduct with DCM, which could also play the role of the carbenium ion initiator, in the presence of DPCs, although moderately (Fig. S22).^68^ These findings indicate that plasmonic DPCs play a critical role by accelerating Cl^−^ generation and the formation of [Al_2_Cl_7_]^−^ and thus AlCl_3_, thereby promoting plasmonic enhanced Lewis acid-mediated hydride transfer and subsequent polymer chain scission.

### Unraveling the role of plasmonic black gold in sunlight-driven LDPE upcycling

**Fig. 5:**
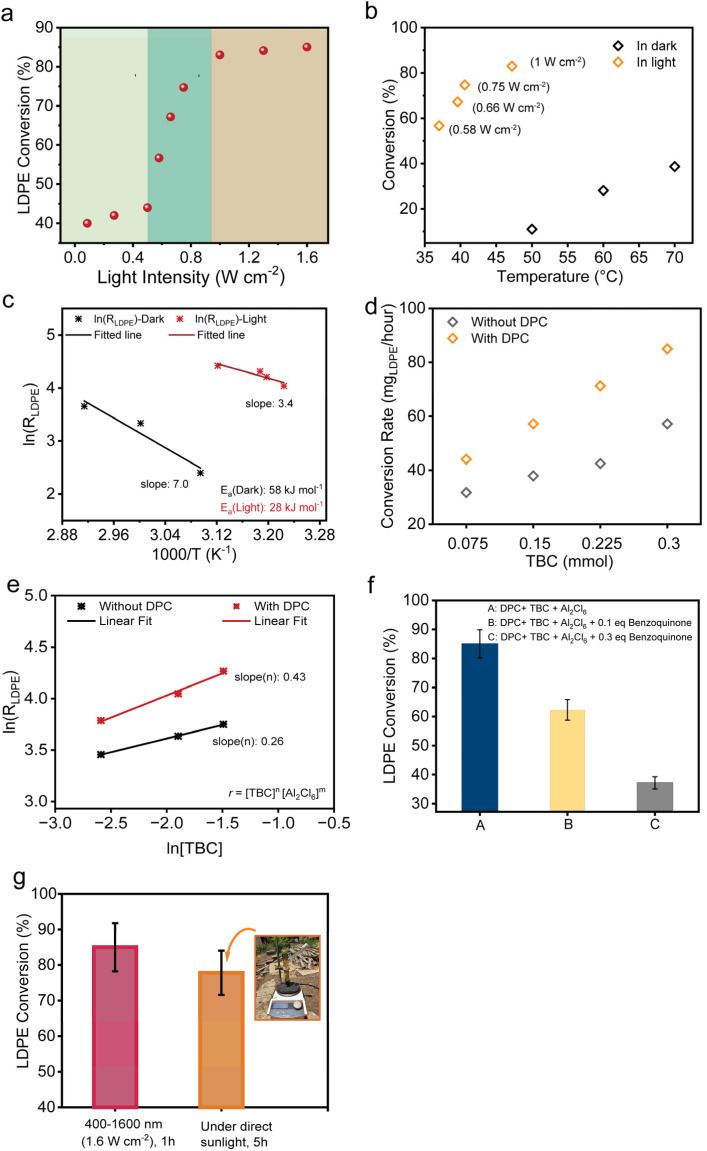
Role of DPCs in plasmonic conversion of LDPE. (a) LDPE conversion rate plotted as a function of light intensity; (b) LDPE conversion profile at different temperatures (in dark) and under light at different intensities; (c) Arrhenius plot for activation energies (*E*_a_) of the plastic upcycling in the dark and light; (d) LDPE conversion with respect to the TBC amount; (e) determination of reaction order as a function of the TBC amount, with and without DPCs under light irradiation (400–1600 nm, 1.6 W cm^−2^); (f) LDPE conversion rate after the addition of electron quencher benzoquinone in the photocatalytic reaction; (g) plasmonic plastic waste upcycling using direct sunlight. Reaction conditions: 0.1 g LDPE, 3 mL DCM, 0.3 mmol Al_2_Cl_6_, 1.0 equivalent TBC, 30 mg DPC, under light, ∼1.6 W cm^−2^ (400–1600 nm), reaction time: 60 min.

To elucidate the contribution of hot electrons generated by plasmonic black gold, we systematically investigated the influence of photon flux on LDPE upcycling under visible to NIR light irradiation. Photocatalytic reactions were carried out at varying light intensities (Fig. 5a and S23). Initially, the reaction rate increased slowly with photon flux; however, at higher light intensities, the rate showed super-linear dependence, indicating multiple electron scattering events from DPCs to adsorbed molecules.^69^ At very high intensities, the rate plateaued, likely due to the saturation of available catalytic adsorption sites on the surface of the black gold.

In order to deconvolute thermal and non-thermal activations, experiments were performed under purely thermal conditions (in the dark) using external heating equivalent to the temperatures attained during light irradiation (Fig. 5b, S12 and S13). In the dark, LDPE conversion remained comparatively low (less than 40%), at respective temperatures. In contrast, the introduction of light irradiation significantly enhanced the reaction (more than 80%), leading to a higher conversion rate (Fig. 5b) indicating that the photothermal effects of plasmonic DPCs alone were not sufficient for catalysis in this case. The Arrhenius analysis indicated the apparent activation energy to be 28 kJ mol^−1^ under light irradiation which was significantly lower as compared to 58 kJ mol^−1^ under dark conditions, again emphasizing the pivotal role of plasmonic non-thermal effects in facilitating polymer breakdown (Fig. 5c). A direct comparison over identical temperature ranges was not feasible because negligible conversion was observed in the dark at temperatures where measurable activity occurs under light irradiation. It should also be noted that the values of activation energies calculated are dramatically lower than the reported activation barriers (163–303 kJ mol^−1^) for polyethylene depolymerization^70^ owing to efficient Lewis acid catalysed polymer cracking.

To further elucidate the role of hot electrons in activating the C–Cl bond, we derived rate expressions *via* a quasi-equilibrium approximation and calculated the reaction orders (*α*_RCl_) across varying concentrations of *tert*-butyl chloride (RCl) (Fig. 5d, e and S24),^60^ under the assumption that C–Cl bond scission is the rate-determining step (RDS). Fig. S24 shows how the reaction orders vary with respect to the reactant molecule, that is, the key for making [Al_2_Cl_7_]^−^ and hence, reactive species AlCl_3_. By assuming that surface coverage of adsorbate ‘*i*’, 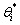 remains constant, the observed increase (Fig. 5e) in *α*_RCl_ (*n* = 0.43) can be ascribed to the dissociative adsorption of RCl being the RDS under photocatalytic conditions.^60^ However, polyolefin cleavage also involves more complex, multi-step pathways beyond dissociative adsorption, including hydride/methyl shifts, carbocation rearrangements, isomerization and β-scission. Thus, the observed kinetics likely reflect a combination of interrelated elementary steps. Under visible-light photolysis, the generation of hot electrons by plasmonic excitation facilitates electron transfer to the σ*-orbital of the C–Cl bond in RCl, promoting its dissociative adsorption on the catalyst surface. This electronic pathway enhances C–Cl bond cleavage, thereby reducing the surface coverage of intact RCl species (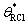) and increasing the reactivity parameter *α*_RCl_ in the presence of light. Such electron transfer can also potentially help in activating the C–H and C–C bonds for the subsequent complex steps. These findings highlight the pivotal role of plasmonic catalysis in accelerating the reaction through electronic pathways that facilitate the dissociative adsorption of RCl, consequently leading to an increased formation of [Al_2_Cl_7_]^−^ under photocatalytic conditions with plasmonic DPCs, as confirmed by ^27^Al NMR.

To directly probe the involvement of hot electrons, benzoquinone, an established electron scavenger,^49^ was introduced in varying concentrations during the photocatalytic reaction (Fig. 5f). A progressive decline in LDPE conversion was observed with the increasing quencher concentration, highlighting that the suppression of hot electron availability adversely affects catalytic performance. This result indicates the significant involvement of hot electrons in driving key elementary steps of the depolymerization mechanism along with the photothermal activation.

### Outdoor demonstration of plasmonic LDPE upcycling under natural sunlight

To evaluate the practical applicability and scalability of this approach, we conducted a small-scale outdoor demonstration using natural sunlight as the energy source (Fig. 5g). 0.1 gram of LDPE particles, 0.3 mmol of Al_2_Cl_6_, 3 mL of DCM, 32.4 µl TBC and 30 mg of DPC were loaded in a reactor. The reactor was directly exposed to natural sunlight, with the incident light intensity periodically recorded between 0.8 and 1 sun, resulting in a maximum reactor temperature of approximately 45 °C (Fig. S25). Remarkably, after 5 h of reaction, LDPE conversion comparable to that achieved under laboratory conditions (16 sun, 1 h) was obtained (Fig. 5g). This efficient performance highlights the practicality of the system, which operates without the need for solar concentrators or external heating infrastructure.

## Conclusions

We have demonstrated a sunlight-only strategy for the catalytic upcycling of plastic waste into value-added hydrocarbons using plasmonic black gold. Under visible-to-NIR irradiation at ambient temperature, our system achieves LDPE conversions exceeding 80% within one hour and yields predominantly C_6_–C_10_ alkanes with >75% selectivity, without external heating, sacrificial reagents, or ionic liquids. Comparative thermal controls, activation energy analysis, and electron scavenging studies, all indicate significant contributions from hot electron-mediated efficient C–Cl cleavage using plasmonic black gold along with photothermal effects.

Quasi-equilibrium microkinetic analysis revealed that plasmonic effects under illumination helped the dissociative adsorption of *tert*-butyl chloride (RCl), as evidenced by an increase in the reaction order. Hot electrons non-thermally excite and cleave the C–Cl bond, lowering 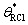 and helping in the formation of [Al_2_Cl_7_]^−^ and hence, more active Lewis-acid AlCl_3_ species. ^27^Al NMR analysis of the reaction mixture at different time intervals revealed that plasmonic effects in DPCs promote the formation of catalytically active AlCl_3_ species, which directly correlates with the observed upcycling rate.

The catalyst remained robust over at least five consecutive cycles, and a proof-of-concept outdoor experiment under one-sun illumination confirmed the practical feasibility of the plasmonic upcycling strategy depicted in this work without solar concentrators or external heaters.

This work represents the first example of plasmon-enabled plastic upcycling powered exclusively by sunlight, merging broadband light harvesting, hot-carrier chemistry, and Lewis acid catalysis into a single, sustainable platform. By eliminating the need for high-temperature reactors, toxic solvents, or hydrogen donors, this approach opens new avenues for low-carbon, on-site conversion of plastic waste into chemical feedstocks and fuels.

## Author contributions

V. P. proposed the research direction, designed the project, and guided the project. S. S. and V. P. designed various experiments. S. S. performed the experiments (synthesis, characterization, and catalysis), assisted by G. S. M. J. assisted with the solution state NMR studies. Data were analyzed by S. S., G. S., and V. P. The overall manuscript was written by G. S., S. S. and V. P. Everyone commented on the manuscript.

## Conflicts of interest

The authors declare no competing interests.

## Supplementary Material

SC-017-D5SC08424E-s001

## Data Availability

The data that support the findings of this work are available within the article and its supplementary information (SI). Supplementary information: experimental details, Fig. S1–S25, Table S1. See DOI: https://doi.org/10.1039/d5sc08424e.
