# Supplementary material for: Solar-driven upcycling of plastic waste using plasmonic black gold
Source: Chem Sci. 2025 Dec 23;17(3):1592–603. doi: 10.1039/d5sc08424e (PMC12777074; doi:10.1039/d5sc08424e)
Supplement: SC-017-D5SC08424E-s001 [file SC-017-D5SC08424E-s001.pdf]

Supplementary Information (SI) for Chemical Science.
This journal is © The Royal Society of Chemistry 2026

Solar-driven upcycling of plastic waste using plasmonic black gold

Saideep Singh,[a#] Gunjan Sharma,[a#] Mamata Joshi,[a] and Vivek Polshettiwar*[a]

[a]Department of Chemical Sciences, Tata Institute of Fundamental Research, Mumbai, 400005 India.

Email: vivekpol@tifr.res.in , # shared 1st authors

EXPERIMENTAL SECTION

Chemicals and materials. All of the chemicals were used as received without additional purification. LDPE [melt index, 25g/10 min (190 °C/2.16 kg; density, 0.925 g/mL)], LLDPE [melt index, 1.0g/10 min (190 °C/2.16 kg; density, 0.918 g/mL)], and HDPE [melt index, 12g/10 min (190 °C/2.16 kg; density, 0.952 g/mL; Mw, 115,828 Da], were purchased from Sigma-Aldrich. Anhydrous aluminium chloride (Al_2Cl_6, 98%), isopentane (iC_5), and tertbutyl Chloride (TBC) were purchased from Sigma-Aldrich. Dichloromethane (DCM, 99.5%) was purchased from SD Fine-Chem Limited. Cyclohexane (99.5%), hydrochloric acid (37 wt%), trichloromethane ($CHCl_3$), and sodium hydroxide (NaOH) are analytical reagents that were purchased from Thermo Fisher Scientific India Pvt. Limited.

Preparation of Polyethylene Particles. Polyethylene (PE) granules (from Sigma Aldrich) were initially subjected to compression at 150 bar using a hydraulic press to achieve uniformity. The compressed polyethylene (2g) was then dissolved into toluene (50 mL) at a temperature range of 80-110 °C under constant stirring at 600 rpm, ensuring complete solubilization. Subsequently, 50 mL dichloromethane (DCM) was introduced into the solution under increased stirring at 1000 rpm while maintaining the same temperature range to facilitate polymer dispersion. The resulting solution was filtered to separate any undissolved particulates, followed by thorough washing with ethanol to eliminate residual toluene. The purified PE material was dried at 80 °C, ensuring solvent removal, before being manually crushed using a mortar and pestle to obtain the final PE particles. For PP, the surgical mask was directly used by cutting it into small pieces without additional cleaning.

Preparation of DPC. DFNS was functionalized with (3-aminopropyl) triethoxysilane using an optimized protocol previously reported to synthesize DFNS-APTS[1,2], followed by grinding 100 mg of DFNS-APTS into a fine powder. The powder was dispersed in 50 mL DI water and sonicated for 15 min before adding 10 mg $HAuCl_4·3H_2O$ (from a 100 mg mL^{-1} stock) and sonicated again for 15 min. After stirring for 2 min at 400 rpm, 40 mg $NaBH_4$ in 1 mL DI water was added dropwise, resulting in a color change from light golden to brown and forming Au nuclei (DPC-N) upon stirring for 2 hours. Without isolation, a separate K-gold solution (150 mg $HAuCl_4·3H_2O$ and 280 mg K_2CO_3 in 45 mL DI water) was vortexed for 15 min and added dropwise, followed by dropwise addition of 1 mL ammonium hydroxide (25% v/v) and 20 mL formaldehyde (37 wt% in H_2O). The solution's color shifted from brown to black over 5-10 min, while stirring continued for 60 min. Finally, the solid product was collected by centrifugation, washed thrice each with ethanol and water, and dried in an oven at 80 °C for 10 hours to yield the black gold powder (DPC).

Characterizations of DPC. Scanning transmission electron microscopy (STEM) analysis was carried out using FEI-Tecnai operated at an accelerating voltage of 200 kV. Elemental mapping was carried out using energy-dispersive X-ray spectroscopy (EDS). Samples were prepared by dispersing a small amount of solid powder in ethanol by sonicating for 10 sec and the dispersion was drop-casted onto a holey carbon-coated 200 mesh copper TEM grid. SEM imaging was performed on a ZEISS ULTRA field emission scanning electron microscope. The SEM was operated with an accelerating voltage of 3 to 10 kV. SEM samples were prepared by drop-casting diluted ethanolic suspension of the powder onto the aluminum stub. PXRD patterns were obtained using a Panalytical X'Pert Pro powder X-ray diffractometer with Cu-Kα radiation. UV-DRS measurements were carried out using a JASCO UV-Vis/NIR spectrophotometer.

Procedure for Catalytic Plastic Upcycling. The photocatalysis was done in a batch reactor using a modified Schlenk tube. The Schlenk tube was cleaned with 3x vacuum and 3x argon then argon was flowed through the reactor at room temperature. Under argon flow, polymers (200/100 mg for different studies), and DPC (30 mg) crushed in mortar pestle were added. The degassing of the reactor at room temperature was done under vacuum for 30 min, followed by the addition of Al_2Cl_6 (79.8 mg, 0.3 mmol), 3 mL DCM (99.5 %), tert-butylchloride (32.4 μL, 0.3 mmol) and for selected studies, isopentane (iC_5, 800 mg, 1.29 mL) under argon flow. The reactor was closed and sonicated for 10s. The reactor was irradiated under visible light (400-1600 nm) using a 1000 W Xenon Lamp (~1.6 W cm^{-2}) under magnetic stirring (500 rpm) for different times under argon. After the reaction, the tube reactor was first cooled down to RT.

Although the normal boiling point of dichloromethane (DCM) is ~40 °C at atmospheric pressure, the reaction was carried out in a sealed, gas-tight reactor. Under light irradiation, the internal pressure increased to approximately 1.6 bar, which correspondingly elevates the boiling point of DCM. As a result, DCM remains largely in the liquid phase and does not completely evaporate despite the measured reaction temperature of ~66 °C. This pressure and possible solute induced boiling point elevation ensures the integrity of the reaction medium during irradiation.

Procedure for Upcycling Product Analysis. To quantify the gaseous products, the reactor was cooled to -20 ^{o}C for 10 min and the gas injection was taken into GC installed with alumina GC column for light hydrocarbon analysis (C_1 to C_4 hydrocarbon isomers). After that, 5 mL of cyclohexane was added using a needle without opening to avoid exposure to the environment. The mixture was held for 30 min at -20 ^{o}C. DCM and cyclohexane were miscible at that temperature, so the liquid with hydrocarbon products was partially extracted after letting the solid DPC settle and stored at 4 ^{o}C (solution A1). The process was repeated one more time using cyclohexane (solution A2). After that, cyclohexane (5 mL) was added at -20 ^{o}C, followed by the addition of 5 mL 1M NaOH. The organic phase with hydrocarbon products and aqueous phase with DPC were allowed to settle. The upper organic layer was extracted and stored at 4 ^{o}C (solution B1). The process was repeated one more time using 5 mL cyclohexane (solution B2). To evaluate and quantify liquid products, 0.5 mL of each solution was taken and 5 μL (3.65 mg) of decane was added as internal standard before injection. 0.5 μL of it was then injected into GCMS having HP-

5ms Ultra Inert Column. C_7-C_{30} n-alkane standard hydrocarbon (1 mg/mL, Supelco-49451-U) samples were used to get the response factor of each C_i by obtaining GC peak area relative to known concentration of each C_i (linear C10 due to internal standard was ignored). Quantification of the hydrocarbon C_i products was then carried out and the mass and corrected mass of Ci were calculated as follows:

Obtained Mass of Ci =

$$\Sigma obtained\ peak\ area\ of\ C_i \times \frac{known\ concenration\ of\ Ci\ in\ n - alkane\ standard}{Peak\ area\ of\ Ci\ in\ n - alkane\ standard} \times volume\ of\ extracted\ solution$$

$$Corrected\ Mass\ of\ Ci = Obtained\ Mass\ of\ C_i \times \frac{added\ mass\ of\ internal\ standard}{obtained\ mass\ of\ internal\ standard\ (in\ 0.5\ mL\ solution)}$$

The selectivity of the hydrocarbon Ci (with i-th carbons) was calculated as:

$$\text{Selectivity (C}i\text{, \%)} = \left(\frac{corrected\ mass\ of\ C_i}{(\Sigma corrected\ mass\ of\ C_i)}\right) \times 100\%$$

To get the conversion from the unreacted polymer, toluene (5 mL) was added to the reactor (DPC in aqueous phase) and heated at 80 °C - 110 °C for 15 min under stirring at 1000 rpm, then left still for 5 min. The top layer, having the dissolved unreacted polymers along with toluene, was stored in a beaker, followed by the addition of 10 mL of DCM to separate the polymer. The steps were repeated two more times using additional 5 mL of toluene each time. The mixture was filtered using a Whatman filter paper(11µm) to get the unreacted polymer. The unreacted polymer particles were washed with 10 mL DCM (x3) and 10 mL EtOH (x3) and dried in an oven at 80 °C.

$$\text{Conversion} = \left(1 - \frac{mass\ of\ the\ residual\ polymer}{initial\ mass\ of\ polymer}\right) \times 100\%$$

The bottom water layer was first neutralized with 1M HCl, followed by filtered using a Whatman filter paper, and by washing with water (x2) and ethanol (x2) to retrieve the used DPC.

^{27}Al and ^{1}H Solution state NMR. NMR spectroscopic measurements were done using Varian 600 MHz spectrometer at the NMR facility, TIFR Mumbai. All the measurements were carried out using CD_2Cl_2 as deuterated solvents, chemical shifts δ are reported in ppm (parts per million). 0.6 mL of the aliquots from the reaction mixture, along with 0.12 mL CD_2Cl_2, was added to the NMR tube for the experiment. The 1M $Al(NO_3)_3$ aqueous solution peaks (in D_2O) were used as an

internal reference, and chemical shifts are expressed relative to Aluminum nitrate (1M $Al(NO_3)_3$, $\delta = 0$ ppm). The parameters used for the NMR spectroscopy were d1 delay = 2 secs, PW90 = 12.5 µs, transmitter power tpwr = 60 dB, spectral width = 242 ppm, number of scans = 256. The d1 delay was fixed at 2 secs on the basis of the results from the inversion recovery experiment. All 1D experiments on the Varian 600 MHz were carried out using the standard Varian 's2pul' pulse sequence. For ^1H NMR, chemical shifts are expressed relative to TMS ($\delta = 0$ ppm). The parameters used for the NMR spectroscopy were d1 delay = 2 secs, PW90 = 5.7 µs, transmitter power tpwr = 57 dB, spectral width = 14.47 ppm, number of scans = 128 and d1= 2 secs.

^{13}C Solid and Solution state NMR. ^{13}C solid state NMR experimental data were collected using the pulse sequence on a JEOL spectrometer (Model: JNM-ECZ600R, JEOL Ltd., Japan) equipped with a 1.0 mm double-resonance (HX) fast MAS probe operating at 599.6 MHz (^1H). A spinning frequency of 12.5 MHz was used. The parameters used for the NMR spectroscopy were: Acquisition delay: 20.52 µs, relaxation delay: 5 secs, MAS spin rate: 12.5 MHz, X90: 2.5 µs, Dwell: 8.0 µs, Number of scans = 256.

^{13}C solution state NMR was done using Varian 600 MHz spectrometer at the NMR facility, TIFR Mumbai after drying the extracted product in cyclohexane under vacuum at 25°C followed by addition of 0.5 mL $CDCl_3$ as the deuterated solvent for locking.

Diffuse Reflectance Infrared Fourier Transform Spectroscopy (DRIFTS) Studies: DRIFT studies were conducted using a JASCO FT/IR-4700 instrument with 10 scans and a resolution of 4 cm^{-1}. 0.3 mmol Al_2Cl_6, 0.3 mmol TBC in 3 mL DCM, stirred in RT for 15 mins for the final spectra. Approximately 60 µL of the above reaction mixture was placed over a stainless steel cup capped with a KBr pellet for spectral measurement.

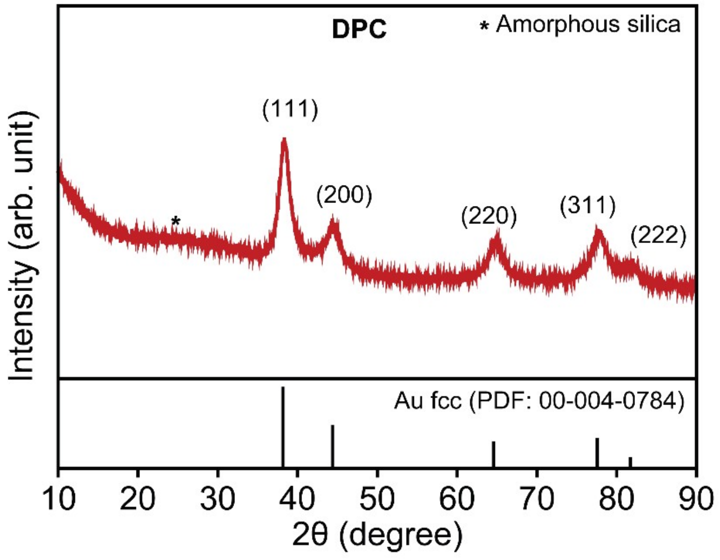

Figure S1. Powder X-ray diffraction pattern of DPC showing crystalline Au.

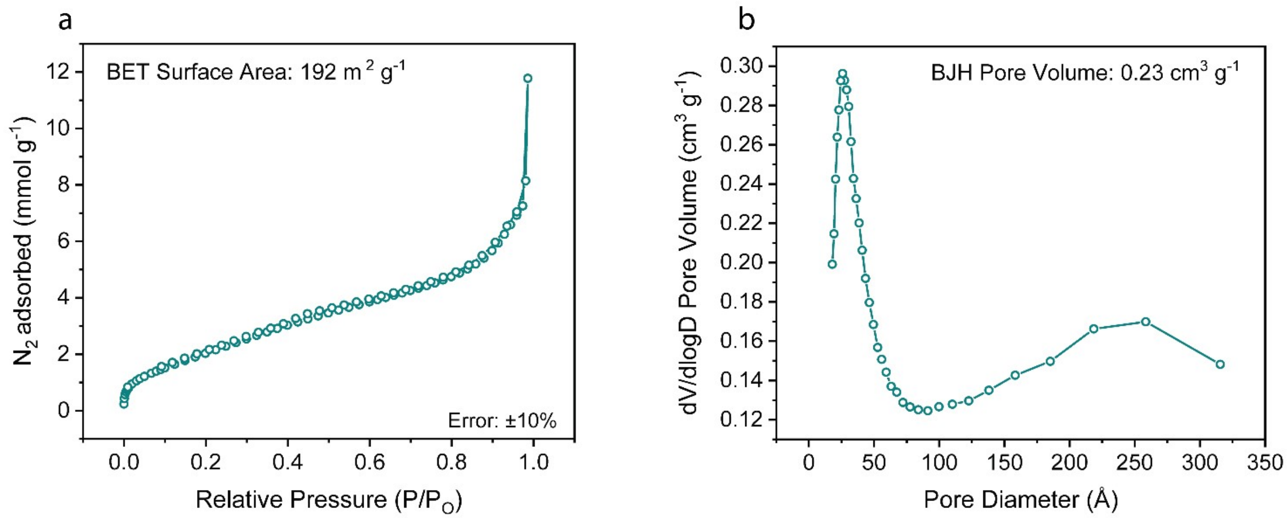

Figure S2. N_2 sorption isotherm and corresponding pore size distribution for DPC.

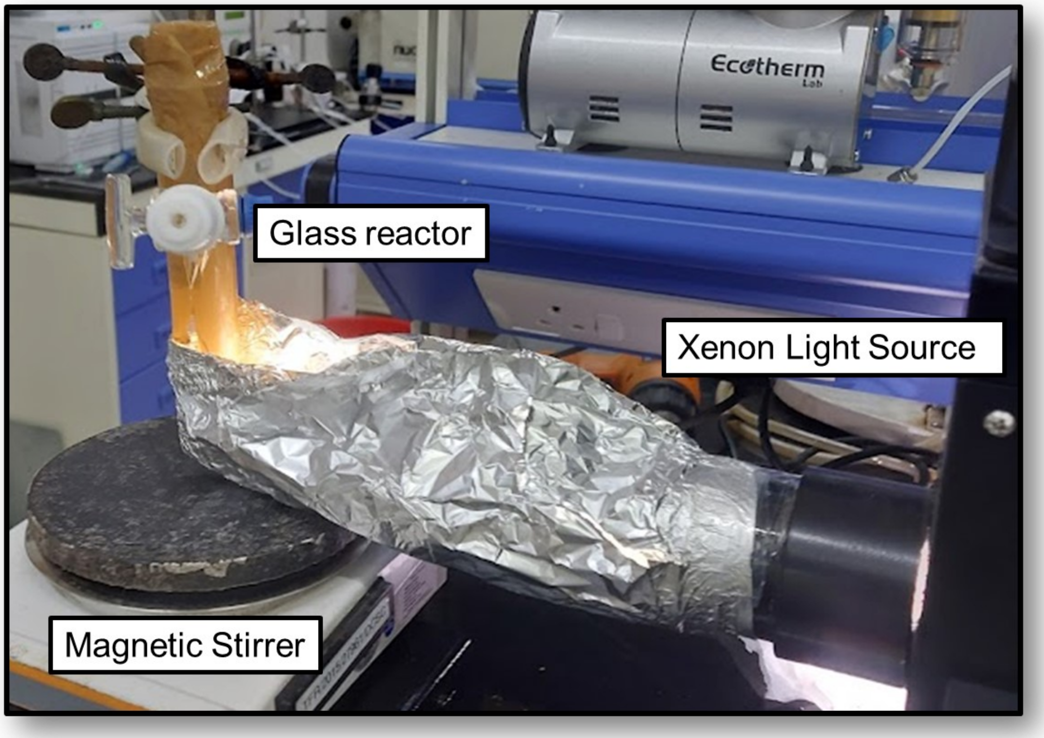

Figure S3. Photograph of the photocatalytic reactor with 0.2 g polyolefin , 3 mL DCM, and 1.29 mL iC$_5$, 0.3 mmol TBC, 0.3 mmol Al_2Cl_6 and 30 mg DPC and under light irradiation ~1.6 W cm^{-2} (400- 1600 nm).

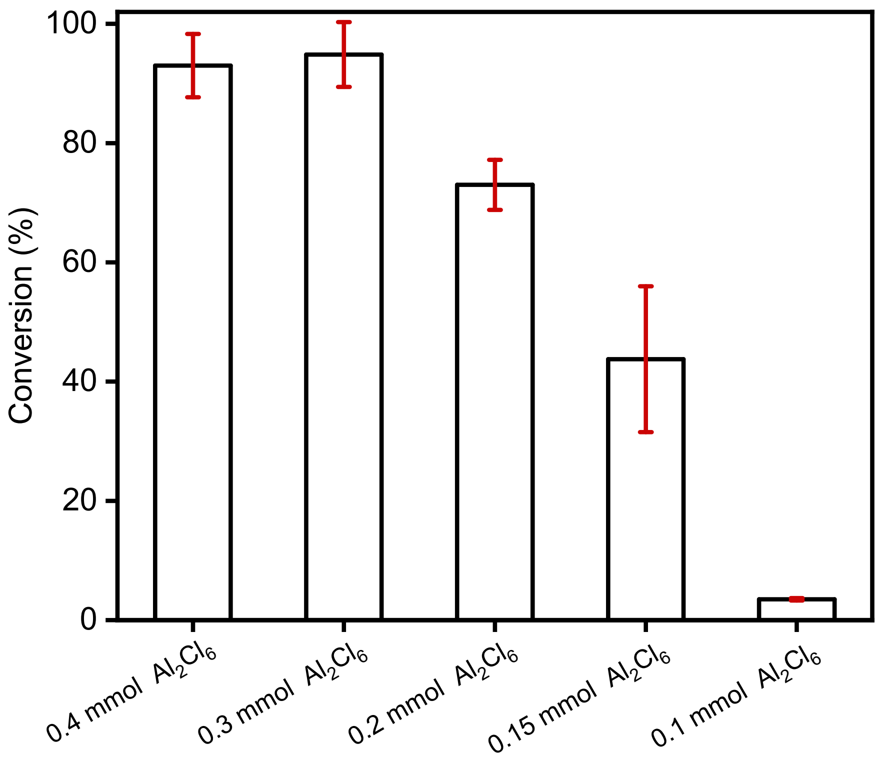

Figure S4. Optimization of the amount of Al_2Cl_6. Reaction Condition: 0.2 g PP, 3 mL DCM, and 1.29 mL iC_5, 1.0 equivalent TBC (with respect to Al_2Cl_6), 30 mg DPC, under light, ~1.6 W cm^{-2}, (400- 1600 nm) for 6 h.

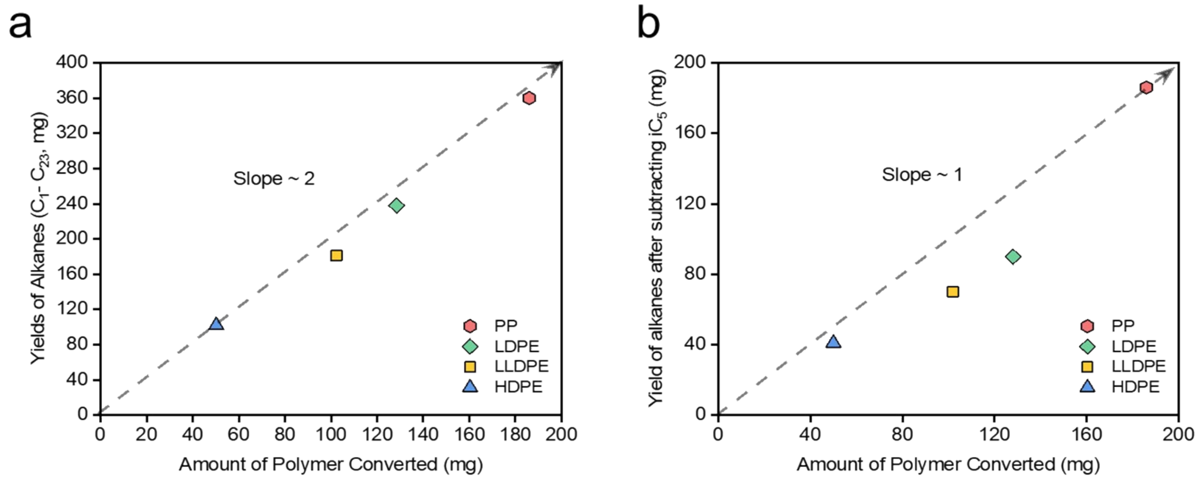

Figure S5. (a) The corresponding total mass of alkanes (C_1 to C_{23}) is plotted against the amount of polymer converted, and (b) after subtracting iC_5 from each C_i. Reaction conditions: 0.2 g polymer, 3 mL DCM, 1.29 mL iC_5, 0.3 mmol Al_2Cl_6, 0.3 mmol TBC, 30 mg DPC, under light, ~1.6 W cm^{-2} (400-1600 nm), 6h.

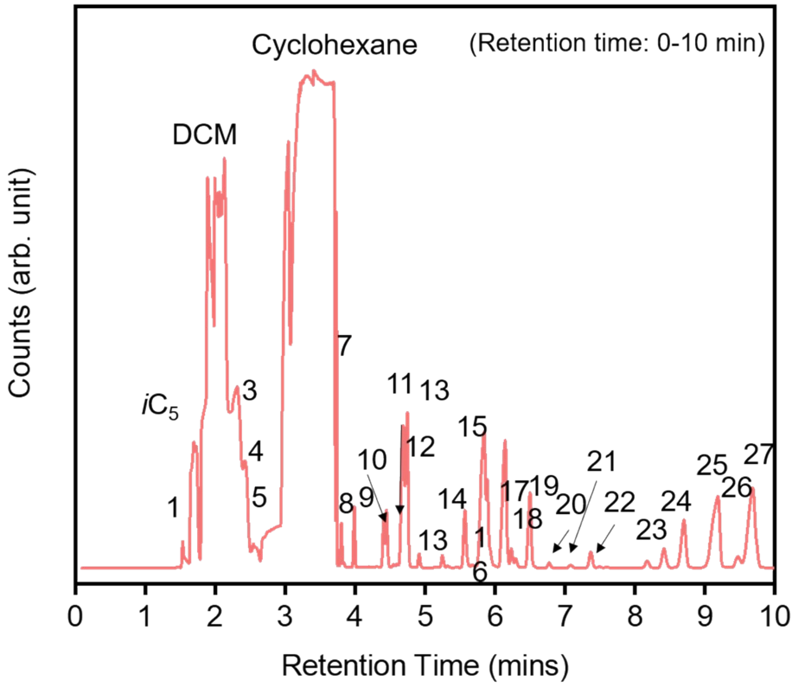

Peak No.	Product Structure	NIST Probability (%)
1	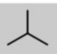	69
2	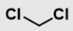	85
3	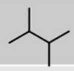	42
4	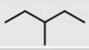	57
5	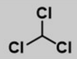	92
6	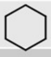	43
7	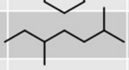	22
8	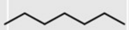	75
9	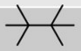	44
10	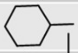	73
11	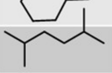	68
12	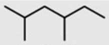	53
13	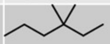	45
14	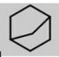	54
15	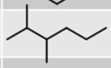	49
16	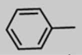	37
17	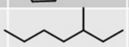	55
18	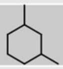	39
19	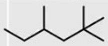	53
20	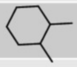	25
21	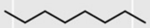	54
22	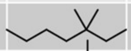	29
23	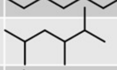	41
24	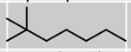	41
25	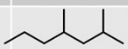	34
26	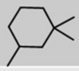	45
27	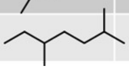	48

Figure S6. GC signals at the retention time from 0–10 min, identified by GC-MS, showing the liquid alkanes from the photocatalytic PP/iC_5 upcycling along with the NIST probability for each species (0-10 min).

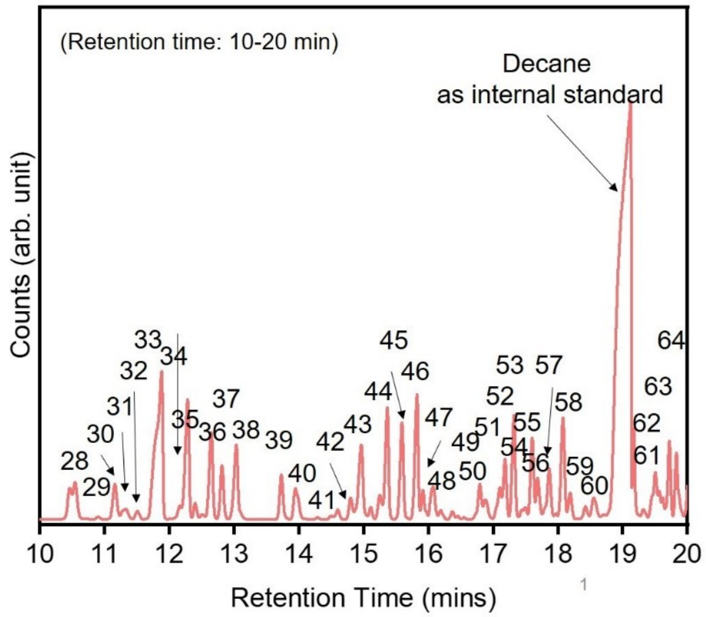

Peak No.	Product Structure	NIST Probability (%)
28	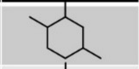	27
29	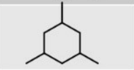	24
30	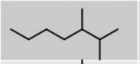	42
31	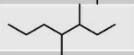	37
32	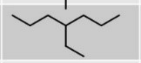	44
33	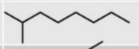	49
34	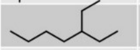	37
35	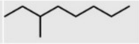	39
36	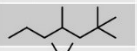	44
37	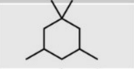	54
38	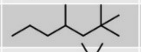	60
39	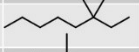	29
40	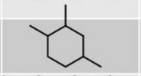	40
41	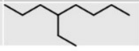	31

Peak No.	Product Structure	NIST Probability (%)
42	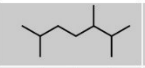	28
43	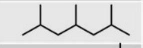	29
44	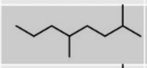	27
45	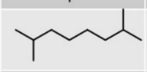	32
46	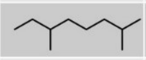	39
47	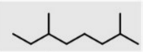	40
48	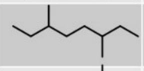	44
49	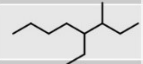	24
50	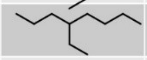	47
51	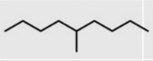	30
52	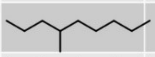	54
53	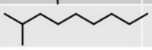	38

Peak No.	Product Structure	NIST Probability (%)
54	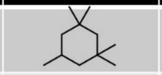	23
55	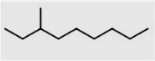	41
56	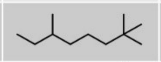	37
57	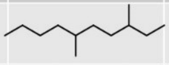	31
58	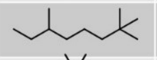	37
59	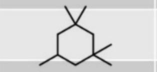	46
60	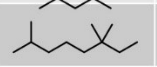	61
61	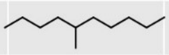	32
62	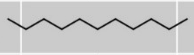	60
63	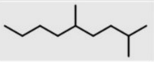	33
64	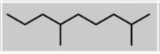	41

Figure S7. GC signals at the retention time from 10–20 min, identified by GC-MS, showing the liquid alkanes from the photocatalytic PP/iC$_5$ upcycling, along with the NIST probability for each species (10-20 min).

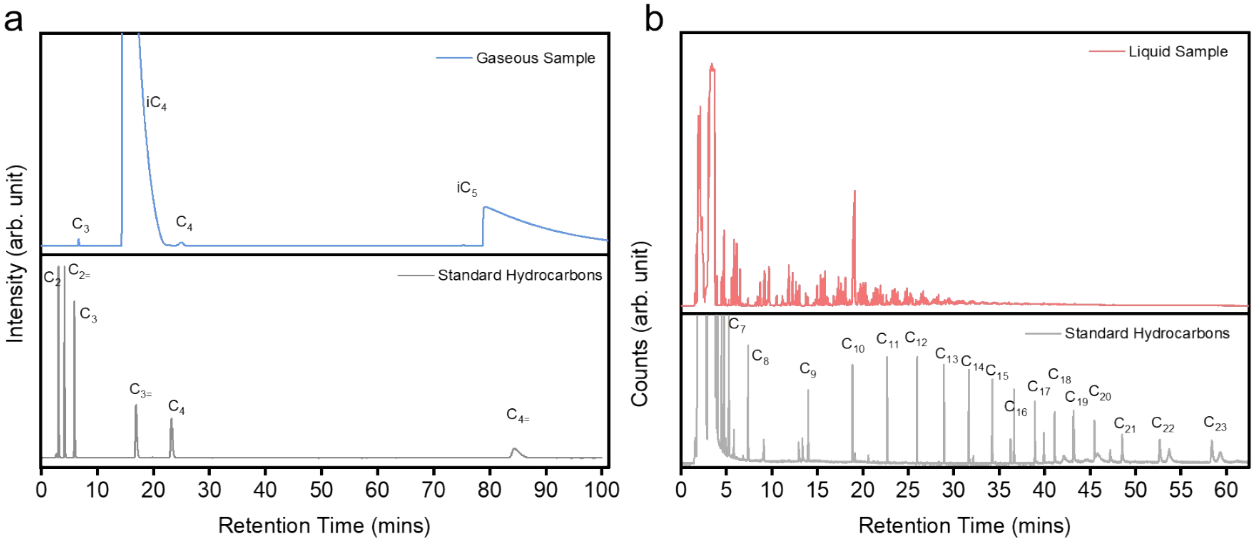

Figure S8. (a) GC analysis of the headspace gaseous sample from the photocatalytic PP/*i*C_5 upcycling, which is calibrated by standard hydrocarbons (ethane, ethene, propane, propene, butane, and butene). (b) GC-MS analysis of hydrocarbon liquid sample from the photocatalytic PP/*i*C_5 upcycling (dissolved in cyclohexane), using n-alkanes as the reference standard.

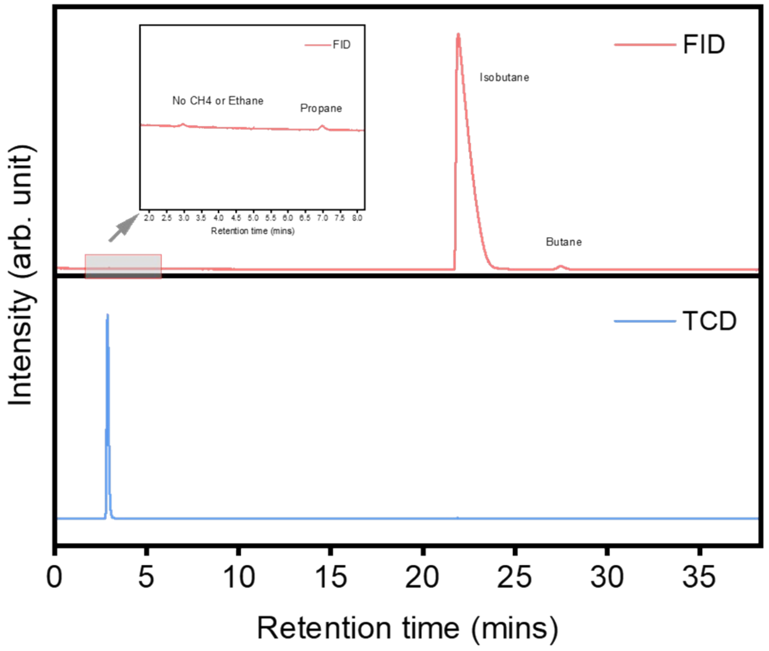

Figure S9. GC analysis of the headspace from the catalytic LDPE/*i*C_5 upcycling showing no sign of CH_4.

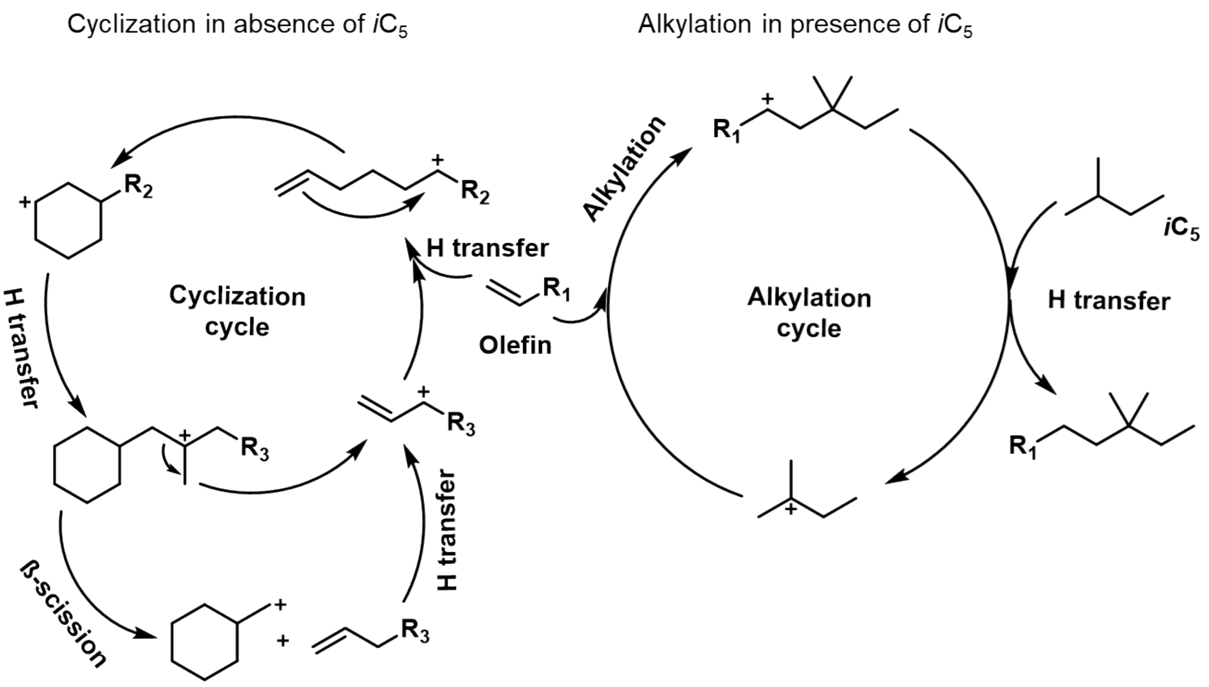

Figure S10. (Left) Proposed reaction pathways for the selective cleavage of LDPE via type-A β-scission of the C–C bond. The cyclo-hydrocarbons are formed by a rapid intramolecular cyclization and break away from the polymer chain via type-A β-scissions. (Right) Proposed reaction mechanism for the alkylation process of a polyolefin with $i\mathrm{C}_5$.

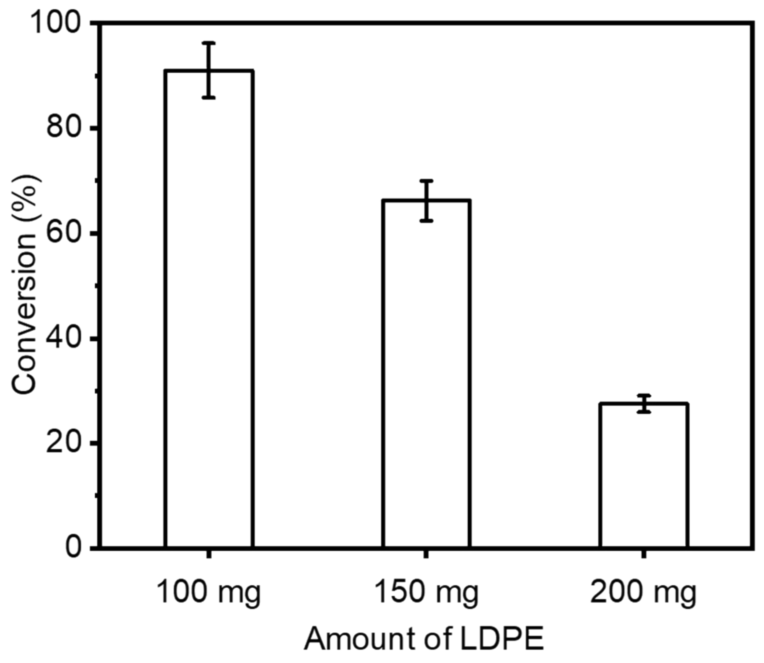

Figure S11. Optimization of the amount of LDPE particles for the photocatalytic conversion without *i*C_5. Reaction Condition: 0.1-0.2 g LDPE, 3 mL DCM, 0.3 mmol Al_2Cl_6, 1.0 equivalent TBC, 30 mg DPC, under light, ~1.6 W cm^{-2} (400-1600 nm), 24h.

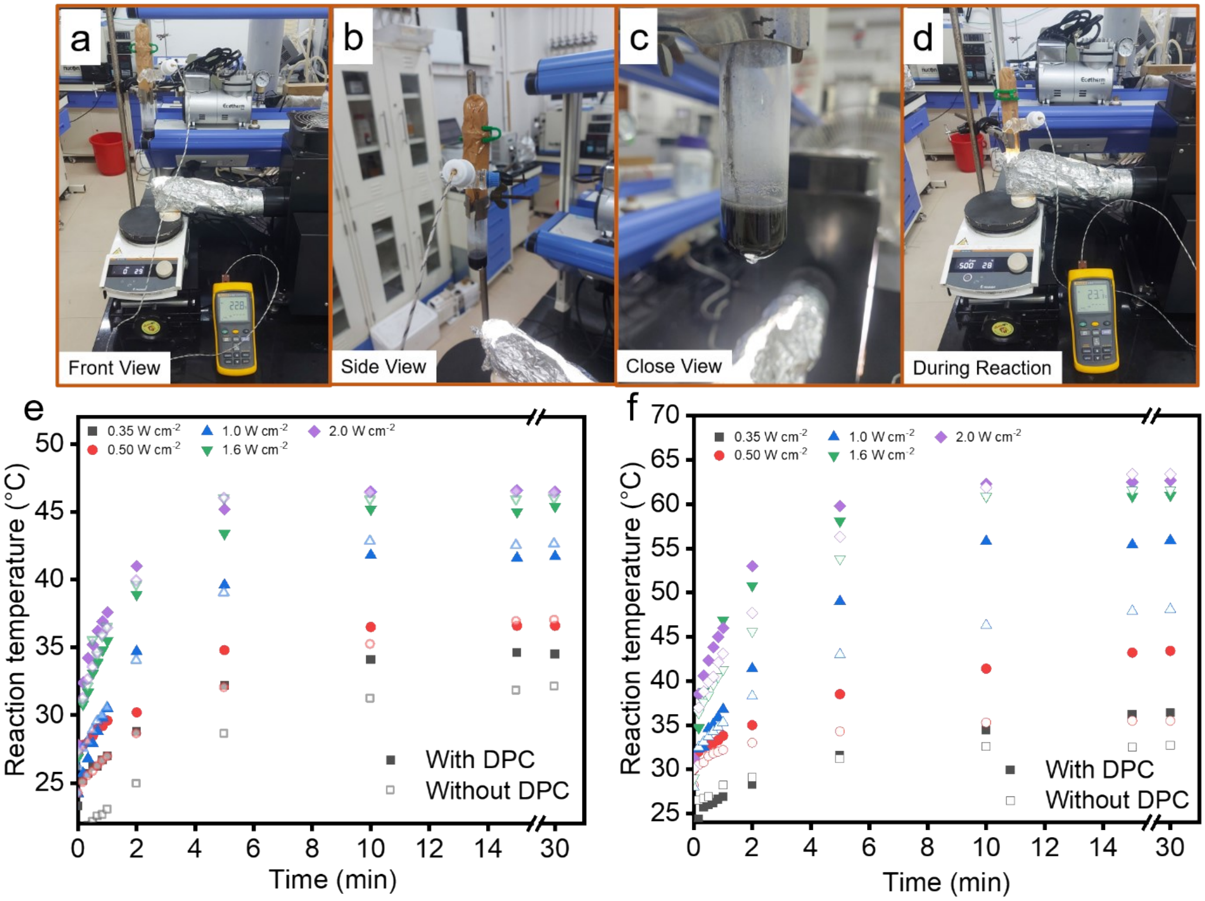

Figure S12. (a) Front, (b) side, (c) close, and (d) during reaction view of in-situ measurement of reaction temperature under light irradiation using a thermocouple. (e) Reaction temperature profile on light irradiation vs irradiation time in the presence and the absence of DPC at different light intensities in the presence of iC_5 (e) using DCM and (f) 1,4-dioxane as solvent. Reaction conditions were as follows: 0.2 g LDPE, 3 mL solvent, 0.3 mmol Al_2Cl_6, 1.0 equivalent TBC, 30 mg DPC, under light 400-1600 nm.

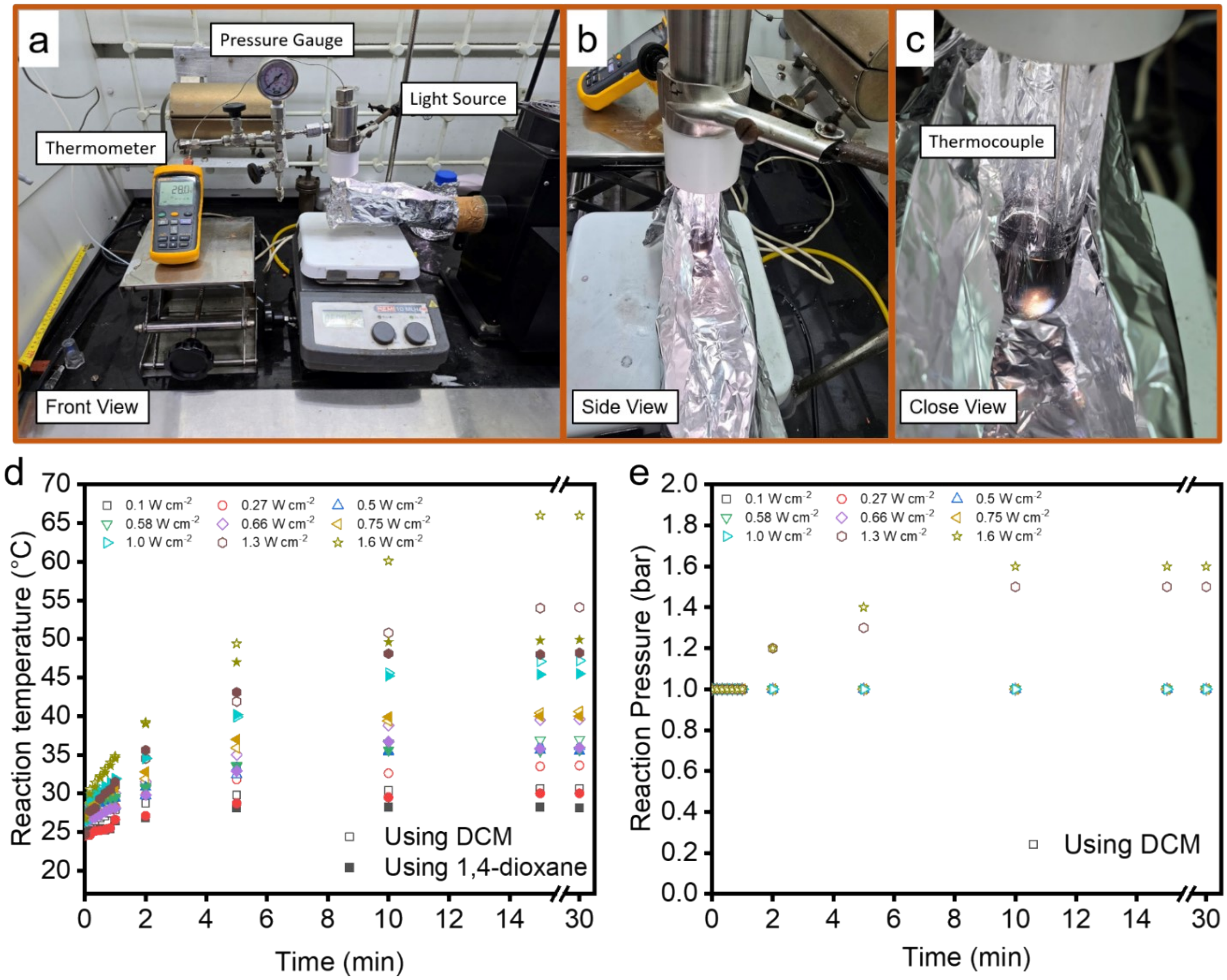

Figure S13. (a) Front, (b) side, and (c) close view of in-situ measurement of reaction temperature under light irradiation using thermocouple. (d) Reaction temperature profile and (e) reaction pressure on light irradiation vs irradiation time in the presence of DPC at different light intensities in the absence of iC_5 in DCM and1,4-dioxane as solvent. Reaction conditions were as follows: 0.2 g LDPE, 3 mL solvent, 0.3 mmol Al_2Cl_6, 1.0 equivalent TBC, 30 mg DPC, under light 400-1600 nm.

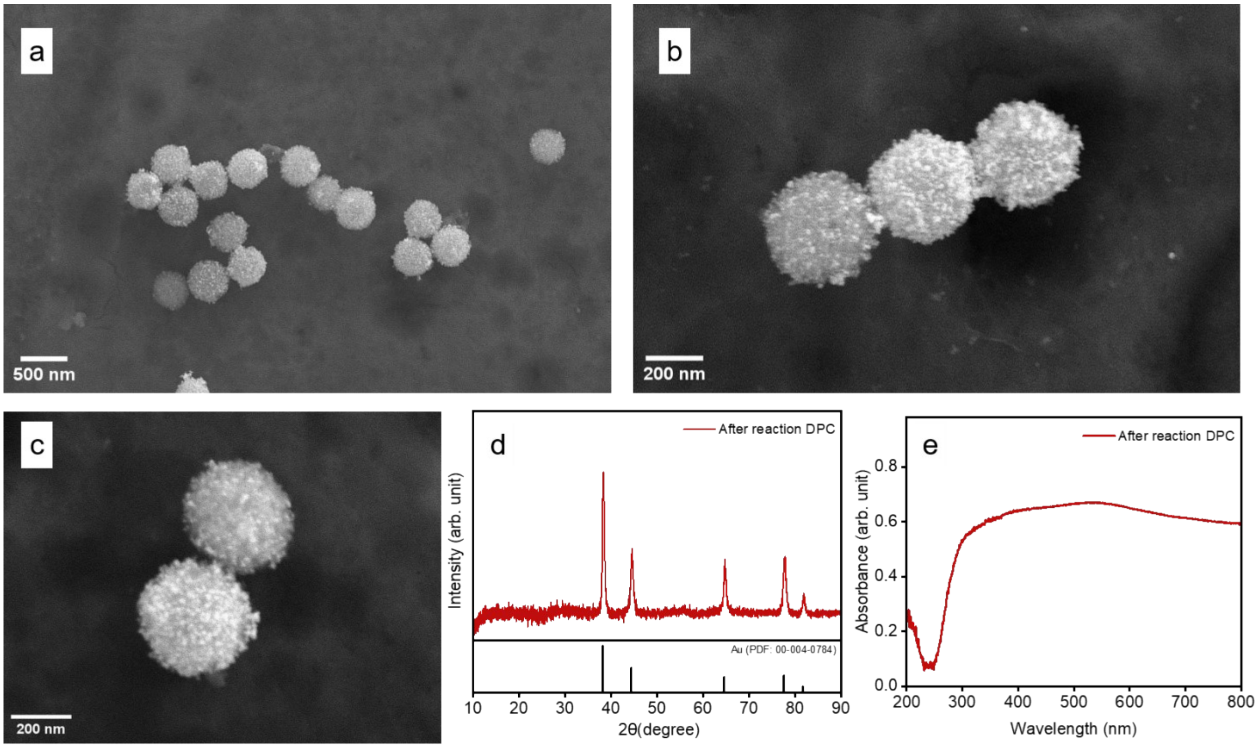

Figure S14. (a-c) Scanning electron microscope images, (d) PXRD patterns, and (e) UV-DRS spectrum of dendritic plasmonic colloidosomes (DPCs) after five times repeated batch tests of catalytic LDPE upcycling reaction.

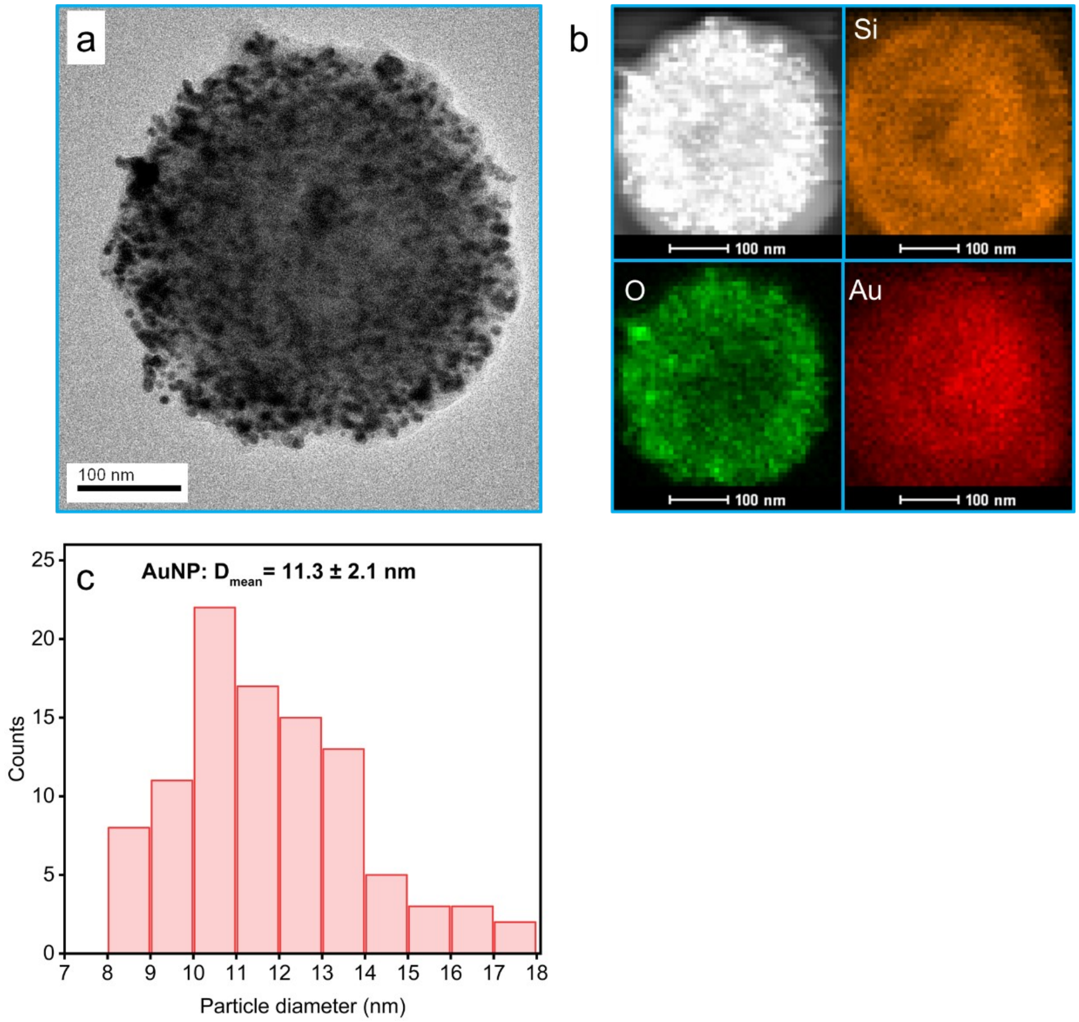

Figure S15. (a) Transmission electron microscope images, (b) STEM−EDS element mapping, and (c) Au NP size distribution (D_{mean}: mean particle size) of dendritic plasmonic colloidosomes (DPCs) after five times repeated batch tests of catalytic LDPE upcycling reaction.

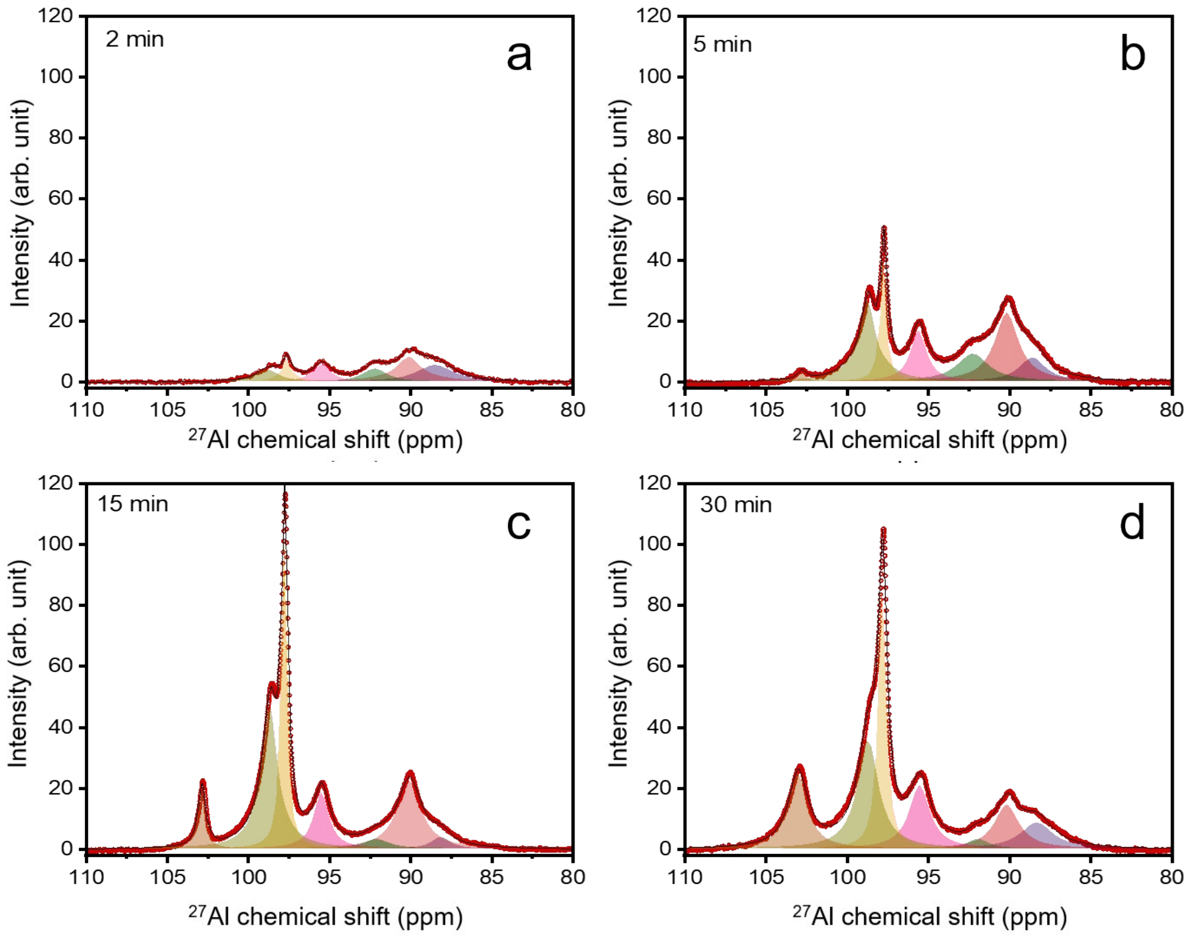

Figure S16. ^{27}Al NMR Peak fittings with different Al intermediates at different time intervals (without DPC) of light irradiation. Reaction conditions were as follows: 0.2 g LDPE, 3 mL DCM, 1.29 mL iC$_5$, 0.3 mmol Al_2Cl_6, 1.0 equivalent TBC, 0 mg DPC, under light, ~1.6 W cm^{-2}(400-1600 nm).

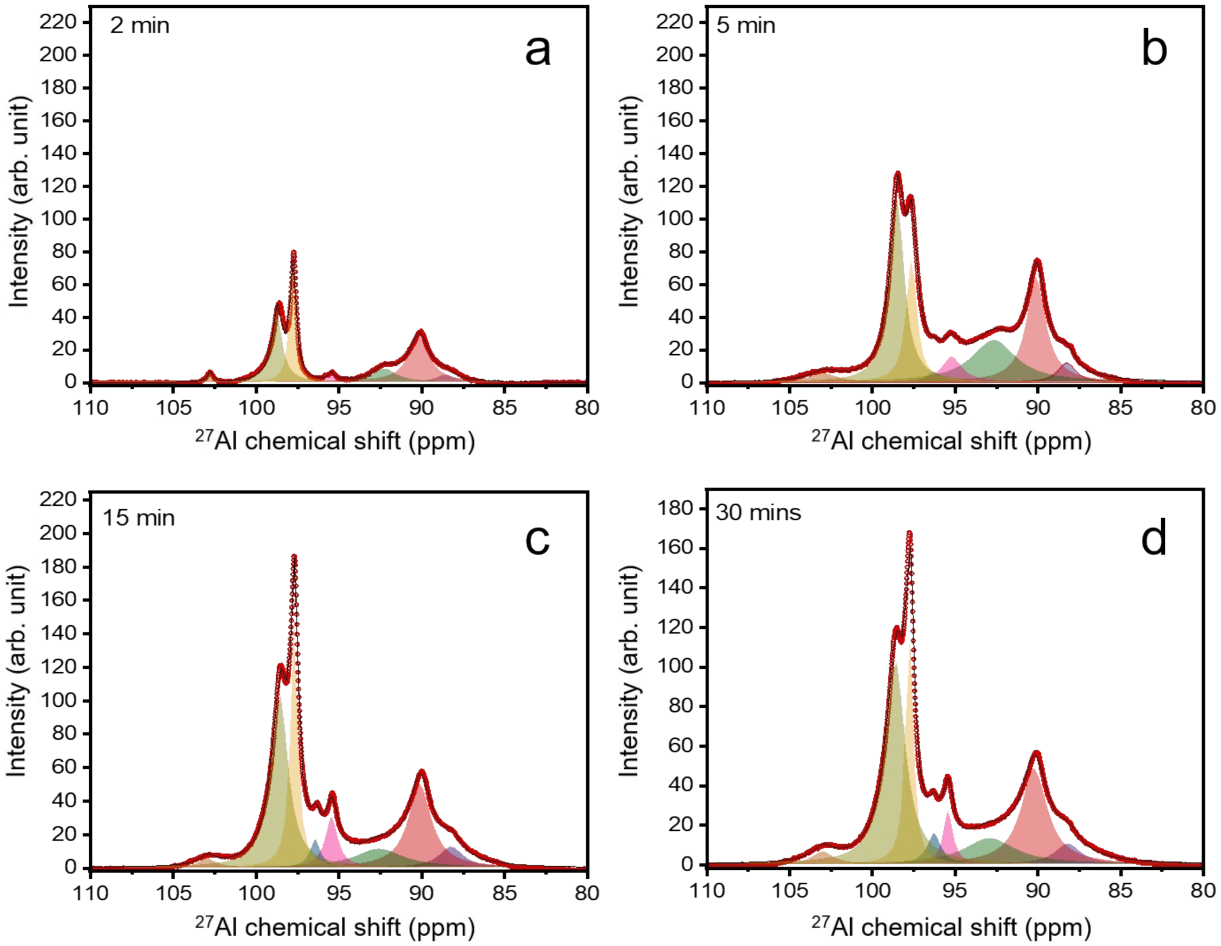

Figure S17. ^{27}Al NMR Peak fittings in different graphs with different Al intermediates at different time intervals (with DPC) of light irradiation. Reaction conditions were as follows: 0.2 g LDPE, 3 mL DCM, 1.29 mL iC_5, 0.3 mmol Al_2Cl_6, 1.0 equivalent TBC, 30 mg DPC, under light, ~1.6 W cm^{-2}(400-1600 nm).

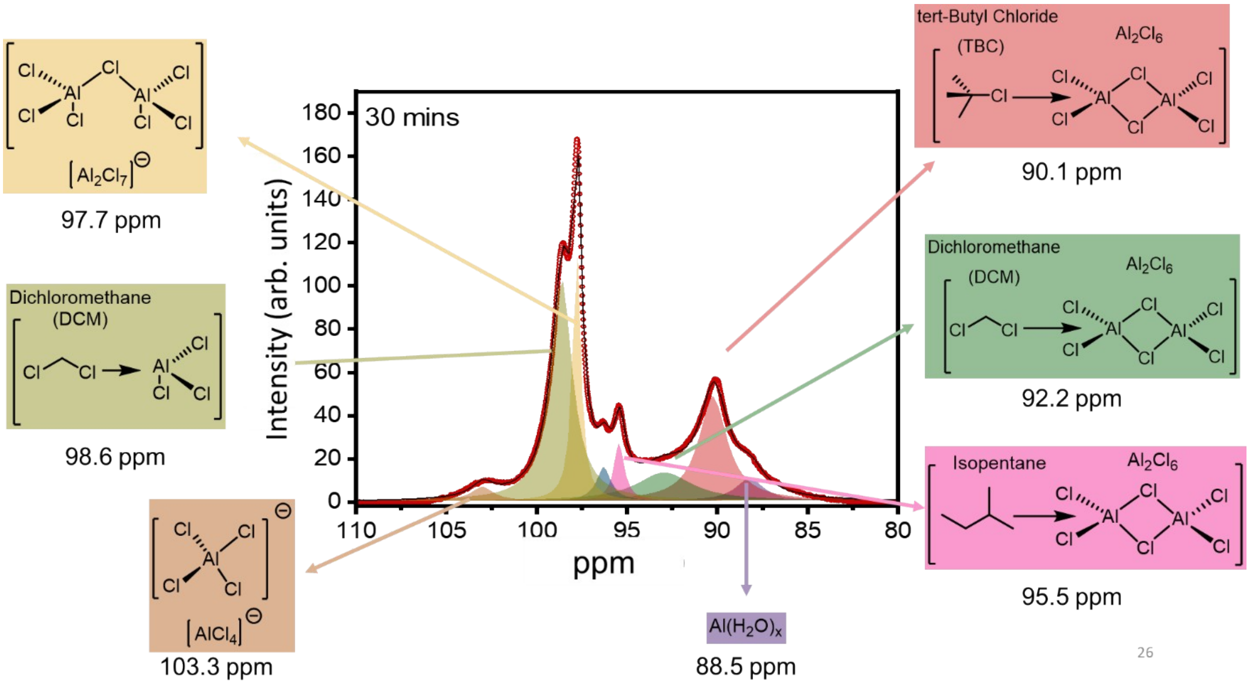

Figure S18. Peak identifications of each Al species of the ^{27}Al solution state NMR based literature previously reported for $[Al_2Cl_7]^-$, $[AlCl_4]^-$ and Aluminium adducts.(ref 3-4)

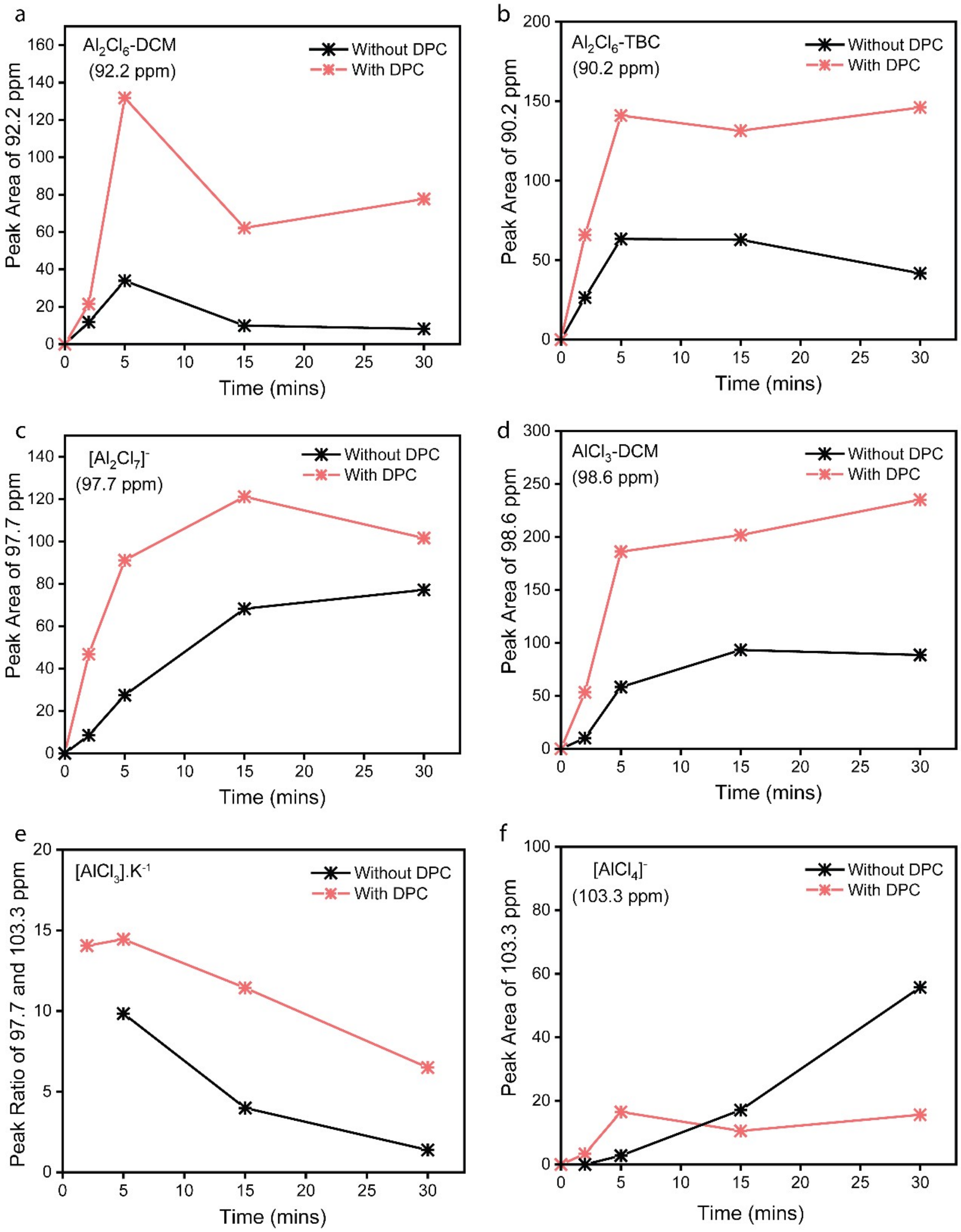

Figure S19. Comparison of the peak area of each Al reactive intermediate (a-f) in the presence and absence of DPC under light irradiation at different time intervals. Reaction conditions were as follows: 0.2 g LDPE, 3 mL DCM, 1.29 mL iC_5, 0.3 mmol Al_2Cl_6, 1.0 equivalent TBC, 30 mg DPC, under light, ~1.6 W cm^{-2}(400-1600 nm).

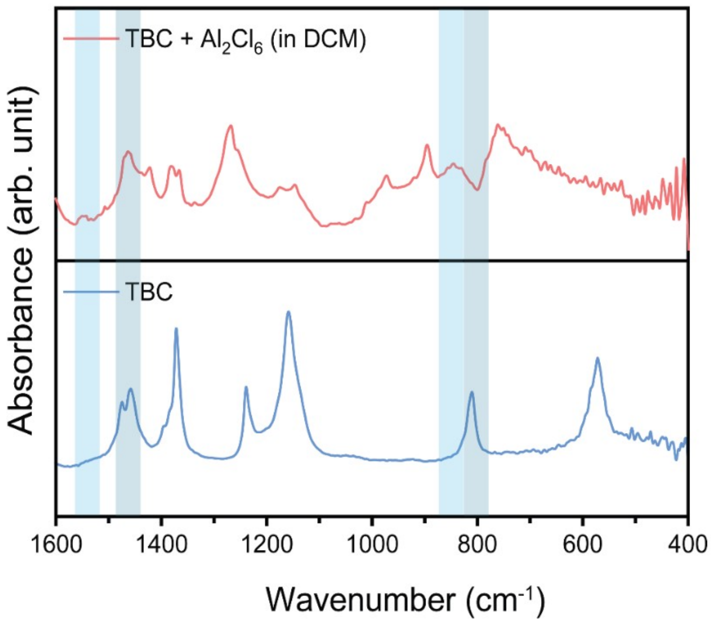

Figure S20. In situ DRIFTS spectra showing the evolution of C-Cl vibrational bands during the interaction of TBC with Al_2Cl_6. (0.3 mmol TBC, 0.3 mmol Al_2Cl_6 stirred in 3 mL DCM for 15 min.

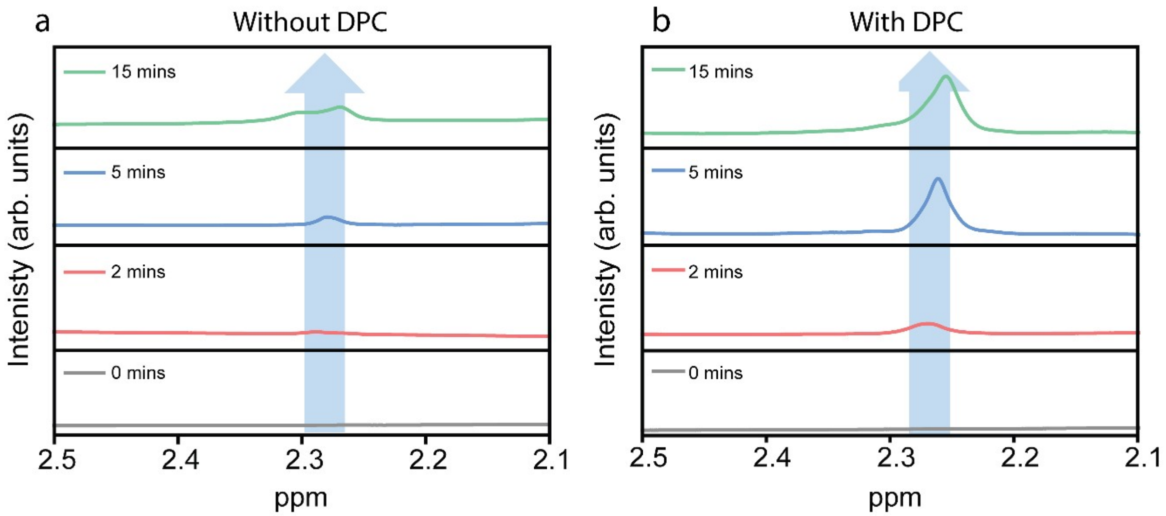

Figure S21. ^{1}H NMR spectra of reaction mixture at different light illumination times with and without DPC showing evolution of isobutane formed after hydride abstraction.

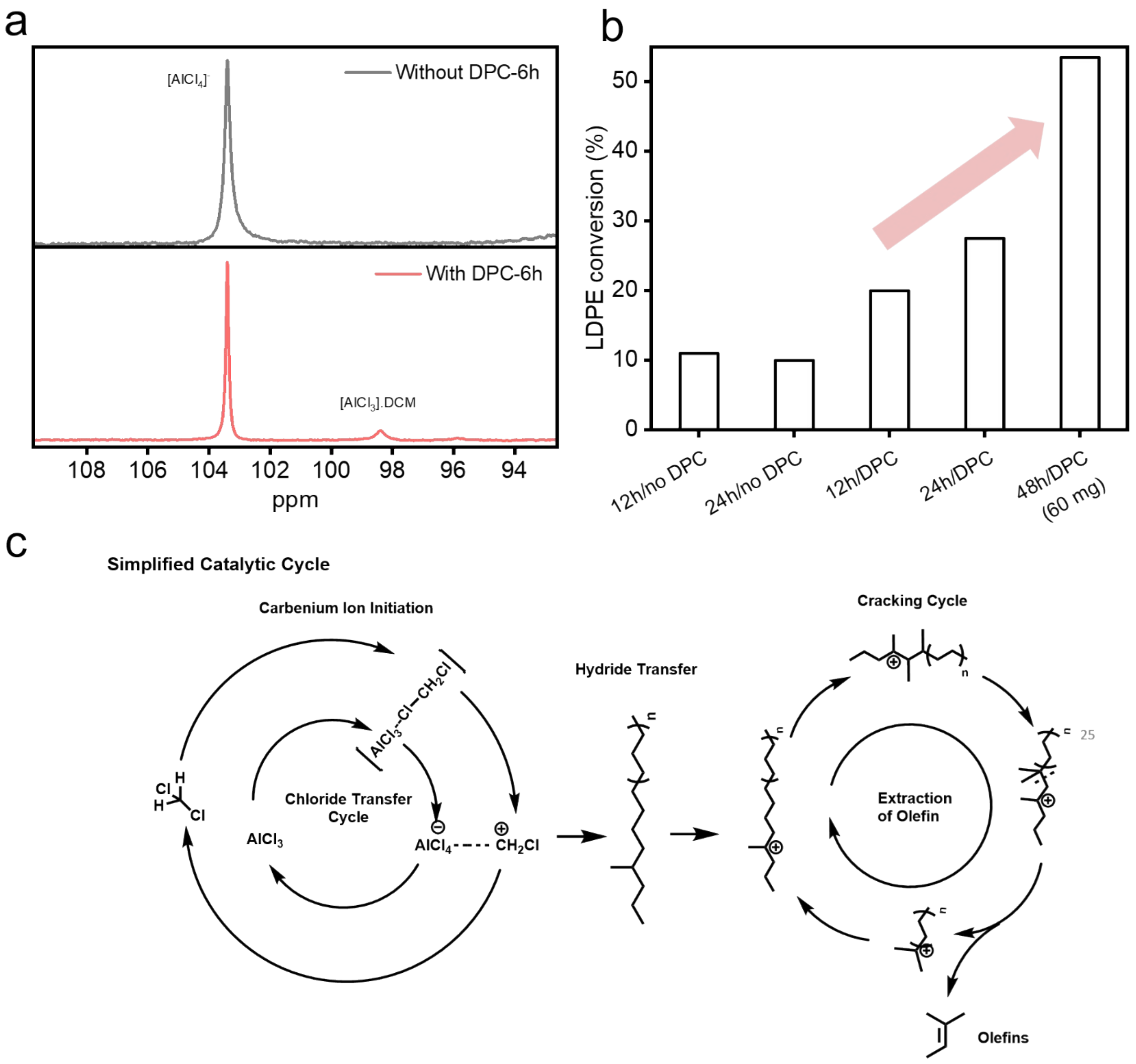

Figure S22. (a) Solution state ^{27}Al NMR after 6h of irradiation showing only $[AlCl_4]^-$ species along with $[AlCl_3]$-DCM species. (b) Increased irradiation time showing increased conversion in presence of DPC showing the role of DPC in regenerating the active species. (c) Regeneration of $AlCl_3$ in the presence of DPC and its proposed mechanism.

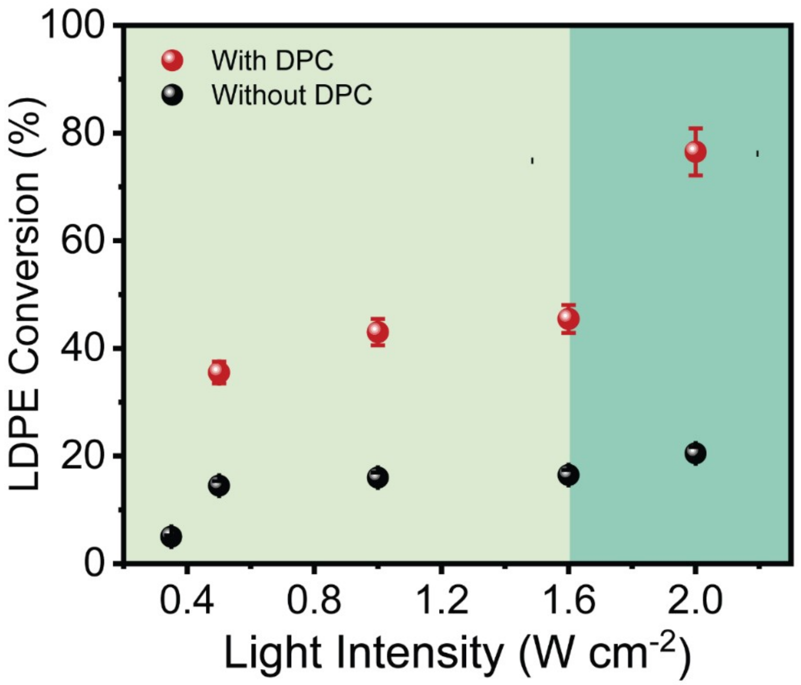

Figure S23. LDPE conversion rate plotted as a function of light intensity. Reaction Conditions: 0.2g LDPE, 3 mL DCM, iC_5 (1.29 mL), 0.3 mmol Al_2Cl_6, 1.0 equivalent TBC, 30 mg DPC, under light, ~1.6 W cm^{-2} (400-1600 nm), reaction time: 60 min

1. $R\text{-}Cl(l) + * \rightleftharpoons R\text{-}Cl^*$ $\qquad k_1[RCl]\,\Theta_* = k_{-1}\,\Theta_{RCl}$

2. $R\text{-}Cl^* + * \rightleftharpoons R^* + Cl^*$ $\qquad rate_{rxn} = k_2\,\Theta_{RCl}\,\Theta_*$

3. $Al_2Cl_6 + * \rightleftharpoons Al_2Cl_6^*$ $\qquad k_3[Al_2Cl_6]\,\Theta_* = k_{-3}\,\Theta_{Al2Cl6}$

4. $Cl^* + Al_2Cl_6^* \rightleftharpoons Al_2Cl_7^* + *$ $\qquad k_4\,\Theta_{Al2Cl6}\,\Theta_{Cl} = k_{-4}\,\Theta_{Al2Cl7}\,\Theta_*$

5. $Al_2Cl_7^* \rightleftharpoons Al_2Cl_7 + *$ $\qquad k_5\,\Theta_{Al2Cl7} = k_{-5}\,[Al_2Cl_7]\,\Theta_*$

6. $R^* \rightleftharpoons R + *$ $\qquad k_6\,\Theta_R = k_{-6}\,[R]\,\Theta_*$

Reaction order $\alpha_{(RCl)} = 1 - 2\theta_{RCl}$ $\qquad$ RCl = Tertbutyl chloride

Figure S24. Proposed mechanism for the TBC cleavage under plasmonic excitation. The asterisk indicates an empty site on the catalyst surface, while A* denotes a site occupied by species A.

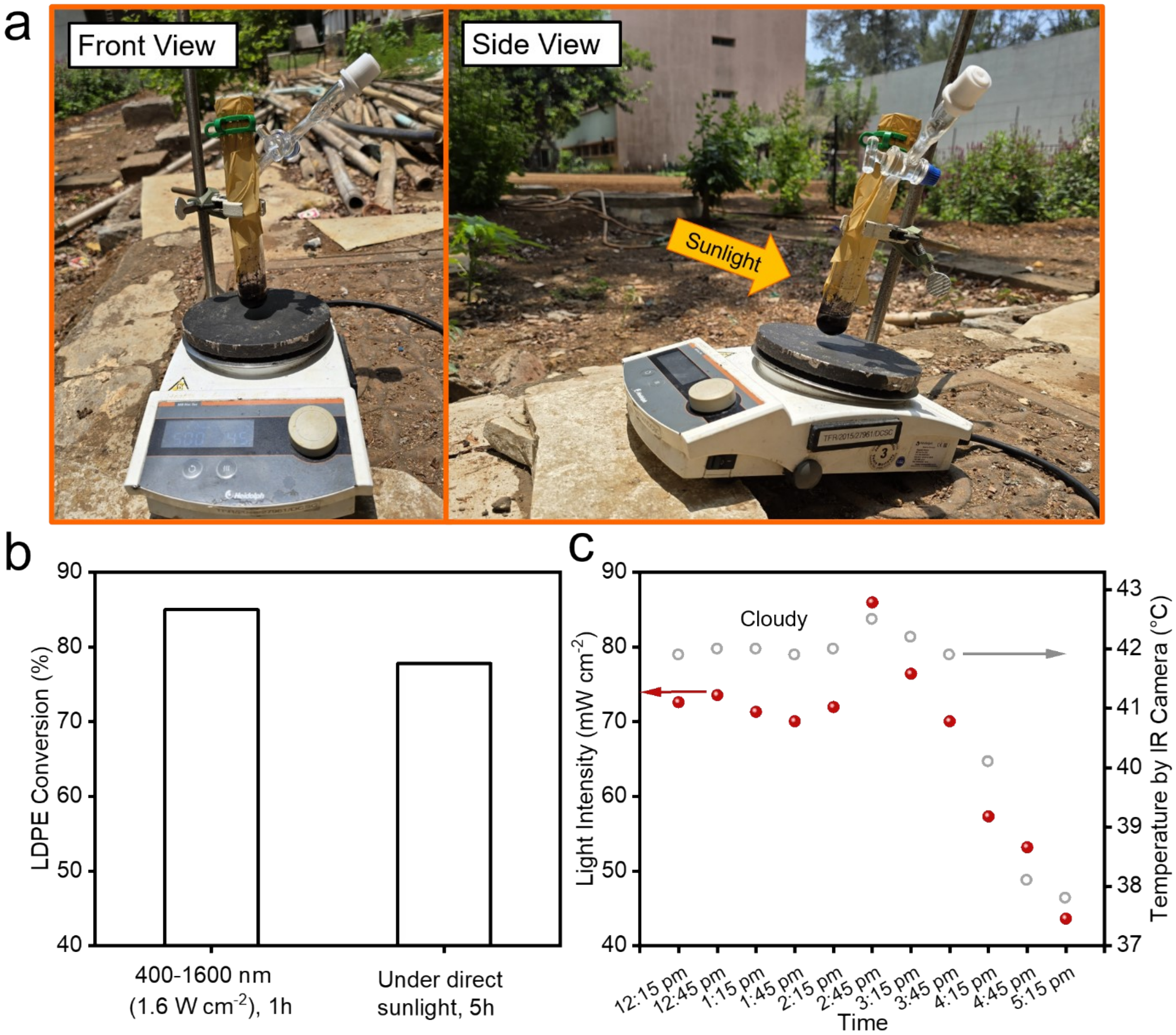

Figure S25. (a) Front and side view of the reactor used for solar catalytic plastic upcycling under direct sunlight with a magnetic stirrer. To ensure maximum sunlight exposure, the reactor was periodically tilted to align with the solar angle. (b) Comparison of LDPE conversion using xenon lamp (1.6 W cm^{-2}, 400-1600 nm) and under direct sunlight. (c) Light intensity and reaction temperature profile of photoreaction under direct sunlight. Reaction Condition: 0.1 g LDPE, 3 mL DCM, 0.3 mmol Al_2Cl_6, 1.0 equivalent TBC, 30 mg DPC, under light, ~1.6 W cm^{-2} (400-1600 nm).

Table S1. Elemental composition of DPCs before and after reaction by SEM-EDS.

Material	Si (wt.%)	O (wt.%)	Au (wt.%)
DPC-Before Reaction	22 ± 3	31 ± 3	48 ± 5
DPC-After Reaction	25 ± 4	44 ± 4	41 ± 8

Supplementary References

1. A. Maity and V. Polshettiwar, Dendritic fibrous nanosilica for catalysis, energy harvesting, carbon dioxide mitigation, drug delivery, and sensing, *ChemSusChem*, **2017**, 10, 3866-3913.
2. A. Maity , R. Belgamwar and V. Polshettiwar, Facile synthesis protocol to tune size, textural properties & fiber density of dendritic fibrous nanosilica (DFNS): applications in catalysis and CO_2 capture, *Nat. Protoc.*, **2019**, 14, 2177-2204.
3. Wu, L., Zhang, R., Zhang, Y., Liu, H., Liu, Z., Xu, C., Meng, X. Aluminum Species and the Synthesis Mechanism of $AlCl_3$−CuCl−Arene Solutions. *Ind. Eng. Chem. Res.* **2021**, *60*, 1155–1163.
4. Nöth, H., Rurländer, R., Wolfgardt, P. An Investigation of $AlCl_3$ Solutions in Ethers by ^{27}Al NMR Spectroscopy. *Z. Nat. B* **1982**, *37*, 29−37.
